# Salt resistance genes revealed by functional metagenomics from brines and moderate-salinity rhizosphere within a hypersaline environment

**DOI:** 10.3389/fmicb.2015.01121

**Published:** 2015-10-13

**Authors:** Salvador Mirete, Merit R. Mora-Ruiz, María Lamprecht-Grandío, Carolina G. de Figueras, Ramon Rosselló-Móra, José E. González-Pastor

**Affiliations:** ^1^Laboratory of Molecular Adaptation, Department of Molecular Evolution, Centro de Astrobiología, Consejo Superior de Investigaciones Científicas – Instituto Nacional de Técnica Aeroespacial, MadridSpain; ^2^Marine Microbiology Group, Department of Ecology and Marine Resources, Mediterranean Institute for Advanced Studies, Consejo Superior de Investigaciones Científicas – Universidad de las Islas Baleares, EsporlesSpain

**Keywords:** functional metagenomics, salt resistance genes, stress response, hypersaline, rhizosphere, brine, saltern, DNA repair

## Abstract

Hypersaline environments are considered one of the most extreme habitats on earth and microorganisms have developed diverse molecular mechanisms of adaptation to withstand these conditions. The present study was aimed at identifying novel genes from the microbial communities of a moderate-salinity rhizosphere and brine from the Es Trenc saltern (Mallorca, Spain), which could confer increased salt resistance to *Escherichia coli*. The microbial diversity assessed by pyrosequencing of 16S rRNA gene libraries revealed the presence of communities that are typical in such environments and the remarkable presence of three bacterial groups never revealed as major components of salt brines. Metagenomic libraries from brine and rhizosphere samples, were transferred to the osmosensitive strain *E. coli* MKH13, and screened for salt resistance. Eleven genes that conferred salt resistance were identified, some encoding for well-known proteins previously related to osmoadaptation such as a glycerol transporter and a proton pump, whereas others encoded proteins not previously related to this function in microorganisms such as DNA/RNA helicases, an endonuclease III (Nth) and hypothetical proteins of unknown function. Furthermore, four of the retrieved genes were cloned and expressed in *Bacillus subtilis* and they also conferred salt resistance to this bacterium, broadening the spectrum of bacterial species in which these genes can function. This is the first report of salt resistance genes recovered from metagenomes of a hypersaline environment.

## Introduction

Life under extreme osmotic pressure in the environment represents a challenge for the vast majority of the microorganisms. Hypersaline habitats such as lakes, salt ponds, and sediments associated with marine ecosystems are considered extreme environments constituted by a discontinuous salinity gradient where salt can reach saturation by evaporation processes (Oren, 2002). These salt-enriched habitats constitute appropriate systems to address questions related to the molecular mechanisms of adaptation to elevated concen trations of NaCl since the native microbial consortia that inhabit these hypersaline environments can grow in the presence of more than 30% (w/v) total salts (Rodriguez-Valera et al., 1985; Antón et al., 2000). Although the predominant salt-adapted organisms belong to halophilic *Archaea* such as the members of the family *Halobacteriaceae*, representatives of *Bacteria* and *Eukarya* can also thrive under these harsh conditions (Oren, 2008).

In general, halophiles adapt to the presence of salt by employing two main strategies to maintain the osmotic balance between the cytoplasm and the surrounding medium: the “salt-in-cytoplasm” strategy and the compatible solute strategy (Galinski, 1995; Sleator and Hill, 2001; Oren, 2008). The ‘salt-in’ strategy is characterized by increasing the salt concentration inside the cell, leading to significant changes in the enzymatic machinery. These include the over-representation of highly acidic amino acids such as aspartate (Asp), and a low proportion of hydrophobic residues that tend to form coil regions instead of helical structures when compared to non-halophile proteins (Paul et al., 2008; Rhodes et al., 2010). Microorganisms that use this strategy include the bacterium *Salinibacter ruber* and also extremely halophilic *Archaea* such as *Halobacterium* sp. whose proteins are very acidic (Oren, 2008). On the other hand, the compatible solute strategy is phylogenetically more widespread than the “salt-in” strategy and consists of the use of osmoprotectants or compatible solutes that do not interfere with the metabolism of the cell. In an initial phase of osmoadaptation using this strategy, high osmolarity conditions can trigger accumulation of K^+^ ions in the cytoplasm, which can eventually lead to salt tolerance as they can serve as intracellular osmoprotectants (Csonka, 1989; Sleator and Hill, 2001). In a secondary response, compatible solutes can act as organic osmoprotectants that are biosynthesized and/or accumulated inside the cell to restore the cell volume and turgor pressure lost during the osmotic stress (Csonka, 1989; Sleator and Hill, 2001). There is a great variety of organic solutes that can act as osmoprotectants, including glycine betaine and glycerol. Some of these solutes are found in specific phylogenetic groups while others are widely distributed in halophilic organisms (Oren, 2008).

The vast majority of the mechanisms of elevated salt resistance and osmoprotection are derived from the knowledge of cultivated microorganisms and their sequenced genomes, thus this information may be biased and may overlook specific strategies of adaptation (Wu et al., 2009). In fact, previous studies using metagenomic sequencing approaches in well-characterized hypersaline environments have revealed novel lineages and genomes from diverse microorganisms without previously cultured representatives (Narasingarao et al., 2012; López-López et al., 2013). Moreover, recent genomic studies on the genus *Halorhodospira* have revealed a combined use of both strategies of salt adaptation (Deole et al., 2013) and through metagenomic analysis an acid-shifted proteome has been described in a hypersaline mat from Guerrero Negro (Kunin et al., 2008). On the basis of these findings, the notion of a correlation between phylogenetic affiliation and the strategy of osmotic adaptation should be revised (Oren, 2013).

Functional metagenomics is a culture independent approach, which is based on the construction of gene libraries using environmental DNA and subsequent functional screening of the resulting clones to search for enzymatic activities. Advantages of this approach include the identification of functional genes during the screening and also that the nucleotide sequences retrieved are not derived from previously sequenced genes, which enables the identification of both novel and known genes (Simon and Daniel, 2009; López-Pérez and Mirete, 2014). Thus, functional metagenomics has recently been used to identify novel genes involved in salt tolerance from microorganisms of a freshwater pond water (Kapardar et al., 2010) and also from the human gut microbiome (Culligan et al., 2012). Nevertheless, to our knowledge a functional metagenomic strategy has not been used to retrieve novel salt resistant genes from microorganisms of hypersaline environments. In this work, we employed this approach to search for salt resistance genes of microorganisms present in two different niches within a solar saltern in the south of Mallorca, Spain: (i) saturated sodium chloride brines, and (ii) moderate-salinity rhizosphere from the halophyte *Arthrocnemum macrostachyum*. To complement the study, the microbial diversity of the brines and the rhizosphere was characterized by amplifying and sequencing the 16S rRNA gene using 454 technology (pyrotagging). The microbial DNA from those samples was also used to construct two small-insert metagenomic libraries which were used to transform the *Escherichia coli* strain MKH13 which is more susceptible to elevated salt concentrations than wild type *E. coli* strains (Haardt et al., 1995). Library screening identified 11 different genes involved in salt resistance, some of which were similar to previously identified genes encoding for proteins conferring salt resistance whereas others encode for proteins that eventually may be related to novel salt resistance mechanisms.

## Materials and Methods

### Bacterial Strains, Media, and Growth Conditions

*Escherichia coli* DH10B (Invitrogen) and MKH13 [MC4100 *Δ(putPA)101 Δ(proP)2 Δ(proU)*; Haardt et al., 1995] strains, and *Bacillus subtilis* PY79 strain (Youngman et al., 1984) were routinely grown in Luria-Bertani (LB) medium (Laboratorios Conda) at 37°C. *E. coli* DH10B was used as a host to maintain and to construct the metagenomic libraries. The growth medium for transformed *E. coli* strains was supplemented with 50 mg ml^-1^ ampicillin (Ap) to maintain the pBluescript SKII (+) plasmid (pSKII^+^), and 100 mg ml^-1^ spectinomycin (Sp) for transformation of *B. subtilis* cells with the pdr111 plasmid. Screening for salt resistance clones and growth curves were carried out in LB medium supplemented with NaCl (Sigma). LB medium also contains NaCl (0.5%), however, the NaCl concentrations mentioned in this study are referred only to the supplemented NaCl.

For the growth curves, cells were cultured overnight in LB broth or LB broth supplemented with 3% NaCl at 37°C, then diluted to an OD_600_ of 0.01 with or without 3% NaCl and 200 ml was transferred to sterile a 96-well micro-titre plate (Starstedt, Inc., Newton, MA, USA) and grown at 37°C for 50 cycles (49 h). OD_600_ was measured every 60 min by using a microplate reader (Tecan Genios, Mannedorf, Switzerland). Non-inoculated wells served as the blank and their values were subtracted from those obtained in inoculated wells. All experiments were carried out in triplicate and the results for each data point were represented as the mean and SEM determined with OriginPro8 software (OriginLab Corporation, Northampton, MA, USA).

### DNA Isolation from Brine and Rhizosphere Samples

Brine and rhizosphere samples used in this study were recovered from the Es Trenc saltern (Mallorca, Spain) in August 2012. Total salinity (%) was determined by refractometry and electric conductivity for brine and rhizosphere samples, respectively, and using three independent replicas. Microbial cells were collected from 400 ml of brine samples by filtration on a 0.22-mm-pore-size membrane filter (Nalgene). The filter was mixed with 5 ml of lysis buffer [100 mM Tris-HCl, 100 mM de EDTA, 100 mM Na_2_HPO_4_ (pH 8.6) and 1% SDS]. The mix was incubated at 65°C with occasional vortex mixing. Samples were centrifuged at 4500 rpm for 5 min at 4°C, and the supernatants were collected. Then, 1.7 ml of NaCl 5 M and 1.7 ml of 10% CTAB were added to the supernatant and then incubated in a 65°C water bath for 10 min with occasional vortex mixing. An equal volume of phenol-chloroform-isoamyl-alcohol (25:24:1; PCIA) was added and centrifuged at 12000 rpm for 15 min at room temperature. The aqueous layer was transferred to a fresh tube and an equal volume of chloroform was added. The mix was then centrifuged at 12000 rpm for 15 min at room temperature. The aqueous layer was removed and transferred to a fresh tube. To precipitate the DNA, 0.6 volumes of isopropanol were added to each tube, stored at room temperature for 1 h and centrifuged at 12000 rpm for 20 min at room temperature. After decanting the supernatant, the pellet was washed with 1 ml of 70% (vol/vol) EtOH and centrifuged at 12000 rpm for 5 min at room temperature. Finally, the pellet was air dried and resuspended in 200 μl of sterile deionized water.

Rhizosphere samples used in this study were obtained from plants of the species *A. macrostachyum*. These samples were kept in 50-mL tubes containing RNA Later (Sigma) and stored at -80°C. In order to extract DNA, the rhizosphere and the soil adhered to the roots were thawed and aseptically processed with the BIO101 FastDNA Spin kit for soil (Qbiogene) and the FastPrep device following to the manufacturer’s recommendations.

### Determination of the Community Structure of the Samples

#### PCR Amplification and 454-Pyrosequencing

16S rRNA gene amplification was performed using bacterial primer pairs GM3 and 630R for *Bacteria* (RB: *Bacteria* in rhizosphere and BB: *Bacteria* in brines), and 21F and 1492R for *Archaea* (RA: *Archaea* in rhizosphere and BA: *Archaea* in brine; Supplementary Table S1) and previously reported conditions (Lane et al., 1985). A five-cycle PCR was performed in a final volume of 25 μL in triplicate to incorporate tags and linker into the amplicon using 1:25 dilution of the original products as templates, and also using the same temperature cycles as for the first PCR. The second PCR was performed using the forward primers GM3-PS (*Bacteria*), 21F-PS (*Archaea*) and the reverse primer 907R-PS (Supplementary Table S1). The products were visualized after electrophoresis in 1% agarose gel run in 1X TAE buffer, at 25 V for 50 min. Two bands were observed, a first of ~1500 bp and the second of ~960 bp. The smaller band was excised and eluted using the Zymoclean^TM^ Gel DNA recovery Kit (Zymo Research, Orange, CA, USA) following the manufacturer’s instructions. The concentration of the barcoded-amplicons was measured with Mass-Ruler Express forward DNA Ladder Mix (Thermo Scientific). Finally, an equimolar mixture of the amplicons was sent to the sequencing company Macrogen, Inc. (Seoul, Korea). The samples were sequenced using 454 GS-FLX+ Titanium technology. Sequences were submitted in the European Nucleotide Archive (ENA) under the Study Accession Number PRJEB9023 (samples ERS696577–80).

#### OTU (Operational Taxonomic Unit) Clustering, Phylogenetic Affiliation, and Selection of OPUs (Operational Phylogenetic Units)

Sequences with <300 nucleotides were removed, and low-quality sequences were trimmed with a window size of 25 and average quality score of 25. No ambiguities and mismatches in reads with primer pairs and barcodes were allowed. Chimeras were removed with the application Chimera Uchime. The trimming process was performed using Mothur software (Schloss et al., 2009). The adequate selected sequences were clustered in operational taxonomic units (OTUs) at 99% using the UCLUST tool in QIIME (Caporaso et al., 2010). We consider one OTU each unique cluster of sequences with identities ≥99%. The longest read of each OTU was selected as representative.

Phylogenetic inference was performed using the ARB software package (Ludwig et al., 2004). Sequences were aligned with SINA aligner (Pruesse et al., 2012), using LTPs115 database (Yarza et al., 2010). Alignments were manually inspected and improved, and sequences were added to the non-redundant SILVA REF115 database (Quast et al., 2013) with the ARB parsimony tool to a default tree. The non-type strain closest relative sequences of an acceptable quality were selected and merged with the LTP115 database. The Neighbor-Joining algorithm was used for the final tree reconstruction, with the Jukes-Cantor correction with *Bacteria* and *Archaea* filter depending Domain, using only almost complete sequences of all reference entries. Representative of each OTU were finally added to the reference tree with the parsimony tool. Sequences were grouped in operational phylogenetic units (OPUs; França et al., 2014) based on the visual inspection of the tree. We consider an OPU as the smallest clade containing one or more amplified sequences affiliating together with reference sequences available in the public repositories. When possible, the OPUs should include a type strain sequence present in the LTP database (Yarza et al., 2010).

#### Ecological Indexes

Operational phylogenetic units were used to calculate rarefaction curves and the Shannon-Wiener (*H*′), Chao 1, and Dominance (*D*) indexes per sample with PAST *v* 3.01 software (Hammer and Harper, 2008).

### Construction of Metagenomic Libraries

The construction of metagenomic libraries and their subsequent amplification was accomplished as previously described (Mirete et al., 2007; González-Pastor and Mirete, 2010). Briefly, the metagenomic DNA was partially digested using Sau3AI, and fragments from 1 to 8 kb were collected directly from a 0.8% low-melting-point agarose gel with the QIAquick extraction gel (QIAGEN) for ligation into the dephosphorylated and BamHI-digested pSKII^+^ vector. DNA (100 ng) excised from the gel was mixed with the vector at a molar ratio of 1:1. Ligation mixtures were incubated overnight at 16°C using T4 DNA ligase (Roche) and used to transform *E. coli* DH10B cells (Invitrogen) by electroporation with a Micropulser (Bio-Rad) according to the manufacturer’s instructions.

### Screening for Salt Resistance

Recombinant plasmids from the metagenomic libraries constructed in *E. coli* DH10B cells were extracted using the Qiaprep Spin Miniprep kit (Qiagen) and ~100 ng of vector were used to transform electrocompetent cells of *E. coli* MKH13. Electrocompetent cells of *E. coli* MKH13 were prepared according to Dower et al. (1988). Cells grown to mid-exponential phase (OD 0.6) were harvested by centrifugation and washed three times with a low salt buffer (1 mM Hepes, pH 7.0). Cells were resuspended in cold 10% glycerol and stored at -80°C.

After electroporation of MKH13 cells, ~5 × 10^4^ transformed cells per amplified library were subsequently screened on LB agar plates supplemented with 50 mg/ml Ap and 3% NaCl, a lethal concentration of salts for MKH13 cells. Plates were then incubated at 37°C for 72 h. To ensure that the resistance phenotype was not due to the presence of chromosomal mutations, the resistant colonies were pooled, their plasmidic DNA was isolated and it was used to transform MKH13 cells, and colonies were selected on LB-Ap plates without 3% NaCl. From each transformation, 100 colonies were patched onto LB-Ap plates containing 3% NaCl. Recombinant plasmids isolated from salt-resistant clones were digested with XhoI and XbaI, to select those which are unique in their restriction patterns.

### *In silico* Analysis of Salt Resistant Clones

The DNA inserts of the plasmids from salt resistant colonies were sequenced on both strands with universal primers M13F and M13R and others for primer walking by using the ABI PRISM dye terminator cycle-sequencing ready-reaction kit (Perkin-Elmer, Waltham, MA, USA) and an ABI PRISM 377 sequencer (Perkin-Elmer), according to the manufacturer’s instructions. Sequences were assembled and analyzed with the Editseq and Seqman programs from the DNAStar package. Prediction of potential open reading frames (ORFs) were conducted using ORF Finder and FGENESB (Solovyev and Salamov, 2011), which are available at the NCBI web page^1^ and www.softberry.com, respectively. The bacterial code was selected, allowing ATG, CTG, GTG, and TTG as alternative start codons for translation to protein sequences. All the predicted ORFs longer than 90 bp were translated and used as queries in BlastP and their putative function was annotated based on their similarities to protein family domains by using Pfam (protein families) available at the European Bioinformatics Institute (EMBL-EBI^2^). Those sequences with an E value more than 0.001 in the BlastP searches and longer than 300 bp were considered as hypothetical. Transmembrane helices were predicted with TMpred^3^

### Cloning of Genes Conferring Salt Resistance

To determine which ORFs were involved in salt resistance in the recombinant plasmids bearing more than one ORF, they were cloned individually in the vector pSKII^+^. Thus, PCR-amplified fragments containing these genes were digested with XhoI/HindIII and XbaI restriction enzymes and ligated into pSKII^+^ digested with the same restriction enzymes. The plasmids obtained were used to transform the MKH13 strain, and growth of the resulting clones was compared with that of the original clone carrying the entire environmental DNA fragment. PCR amplification of the ORFs was carried out using the following reaction mixture: 25 ng of plasmid DNA, 500 μM of each of the four dNTPs, 2.5 U of *Pfu* Ultra DNA polymerase (Stratagene) and 100 nM of each forward and reverse primers (described in Supplementary Table S2A, Supporting information) up to a total volume of 50 μl. The PCR amplification program used was as follows: 1 cycle of 5 min at 94°C, 30 cycles of 30 s at 94°C, 30 s at 52°C, 5 min at 72°C and finally 1 cycle of 10 min at 72°C. PCR amplification products were excised from agarose gels and purified using the Qiaquick Extration Gel kit (Qiagen). Purified PCR products were then digested with the appropriate restriction enzymes (Roche) and ligated into pSKII^+^. To incorporate their native expression sequences (promoters and ribosome binding sites), a region of ~200 bp located upstream of the start codon was also amplified. Some of the ORFs were truncated or the 5′ region was close to the polylinker sequence of the pSKII^+^ vector, and they were subcloned in the same orientation as of the original clone. The *E. coli* genes encoding the endonuclease (*nth*) and the RNA helicase (*rhlE*) were amplified by PCR from DNA of the MKH13 strain (primers are described in Supplementary Table S2B) and similarly subcloned in the pSKII^+^ vector. *E. coli* genomic DNA was isolated using the Wizard Genomic DNA Purification Kit as recommended by the manufacturer (Promega, Madison, WI, USA). The MKH13 strain was transformed with these genes and the growth of the resulting strains was tested by growth experiments carried out on LB-agar supplemented with 3% NaCl.

To assess the salt resistance in *B. subtilis*, the genes were cloned in plasmid pdr111 using the specific primer listed in Supplementary Table S3. This plasmid was a gift from D. Rudner (Harvard Medical School) and derives from pDR66, thus carrying front and back sequences of the *B. subtilis amyE* gene, which encodes an alpha-amylase. It also contains the hyper-SPANK promoter (Phyperspank), which is inducible by IPTG. The recombinant plasmids were then transferred to *B. subtilis* strain PY79 with selection for Sp resistance. pdr111 is not capable of replication in *B. subtilis*, thus the DNA fragment is inserted in the *amyE* locus in the chromosome, the transformants were screened for the absence of amylase activity on starch plates. Briefly, for transformation of *B. subtilis*, cultures grown overnight on LB broth at 30°C were diluted to OD600 nm of 0.08 in 10 ml of the modified competence medium (MCM) and were incubated at 37°C with agitation (200 rpm; Spizizen, 1958). At the onset of stationary phase (OD 600 nm = 1.5–2), 1 mg of the recombinant plasmids were added to 1 ml of the culture. Then, culture was incubated at least 2 h at 37°C and 200 rpm before plating on LB solid medium containing Sp (100 mg ml^-1^). Growth curves were carried out as previously described either in the presence or in the absence of 1 mM IPTG.

### Elemental Quantification of Na^+^ in Resistant Clones

*Escherichia coli* MKH13 carrying the empty vector and recombinant clones were grown aerobically in LB liquid medium containing 50 mg ml^-1^ Ap at 37°C in a shaking incubator, and growth was monitored as optical density at 600 nm (OD_600_). NaCl was added at 6% in early stationary phase to the cultures and grown for one additional hour. Cultures were washed four times extensively with ultrapure MiliQ H_2_O and centrifugation. Washed pellets were lyophilized, pulverized and subsequently the concentration of Na^+^ was measured by inductively coupled plasma spectroscopy-mass spectrometry (ICP-MS) analysis at SIdI (UAM, Madrid). Results were expressed as mg of Na^+^ g^-1^ dry weight of cells. One-way ANOVA and Tukey’s test were used for statistical analysis with OriginPro8 software (OriginLab Corporation, Northampton, MA, USA).

## Results

### Microbial Community Structure of the Brine and Rhizosphere Samples

**FIGURE 1:**
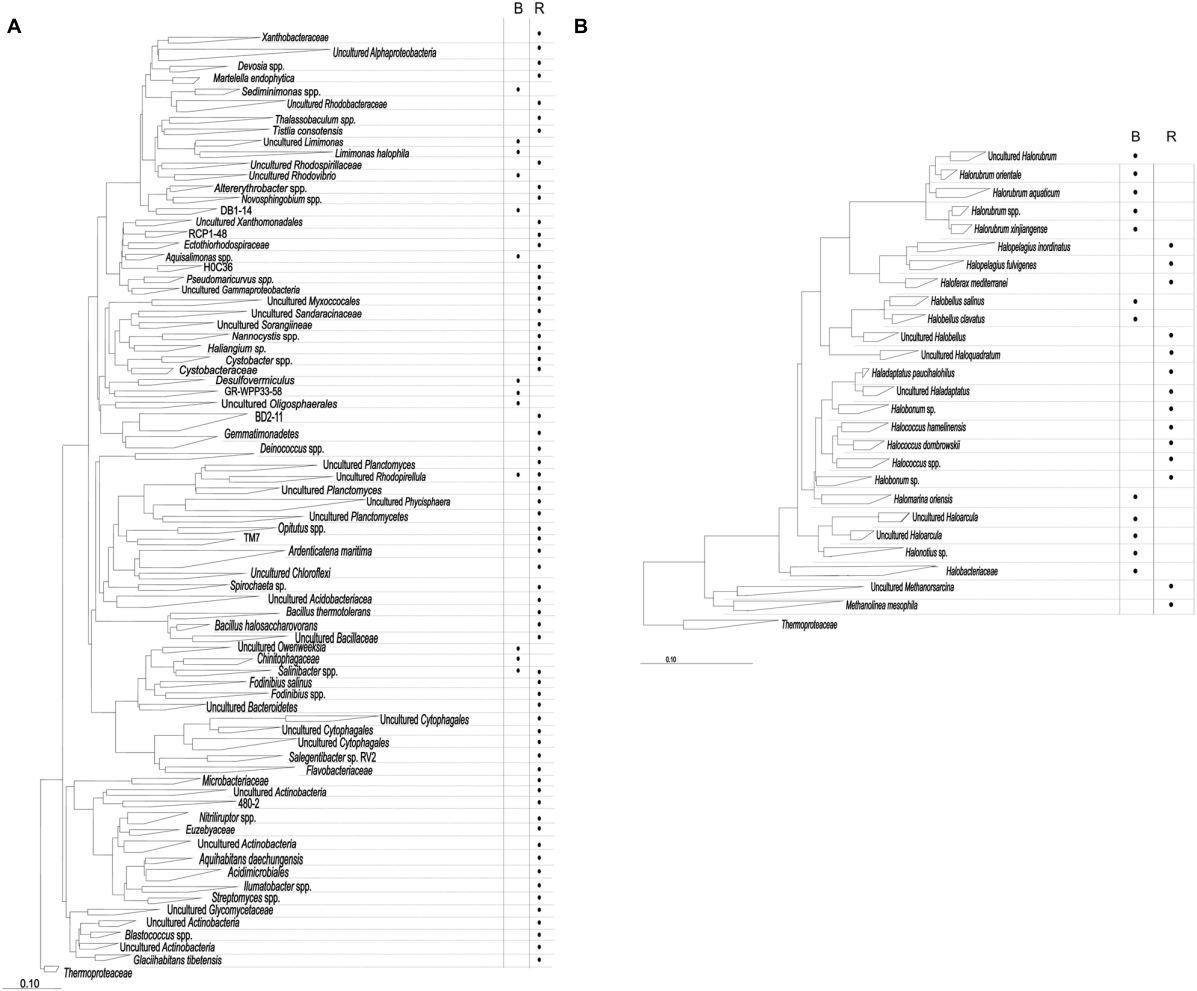
**16S rRNA phylogenetic reconstruction for bacterial **(A)** and archaeal **(B)** sequences.** The presence of OPUs with abundances >0.5% in each sample type (B = brines; R = rhizosphere) is indicated with a dot. Each OPU results from the phylogenetic inference resulting from the parsimony insertion of representatives of each sequence cluster at 99% identity, each representing independent OTUs.

In order to search for genes that could confer increased salt resistance to *E. coli*, we sampled two sites in the hypersaline environment Es Trenc: (i) brine from a crystallizer pond (total salinity of 38.53 ± 0.23%), and (ii) moderate-salinity rhizosphere from the halophyte *A. macrostachyum* (total salinity of 3.28 ± 0.48%). DNA isolated from these samples was used to explore the bacterial and archaeal diversity. 16S rRNA gene sequences were clustered at an identity threshold 99%, resulting in a total of 970 OTUs (Supplementary Table S4) that after the phylogenetic inference produced a total of 226 OPUs, 200 for *Bacteria* and 26 for *Archaea* (**Figure 1**, Supplementary Table S5).

Most bacterial OPUs (187 OPUs*)* were detected only in RB, while BB contained just 13 OPUs, and only two were shared by both samples (OPUs 109 and 144). The sequences were distributed in 16 phyla (**Figure 1A**; Supplementary Table S5). A total of 102 OPUs affiliated with the phylum *Proteobacteria* (47 *Alpha-*, 8 *Beta-*, 30 *Gamma-*, and 17- *Deltaproteobacteria);* 31 with *Actinobacteria*, 27 with *Bacteroidetes* and 17 with *Firmicutes*. The major OPUs in RB were OPU 120 (*Ardenticatenamaritima*, 5.0%), OPU 153 (*Cytophagales*, 3.6%), OPU 125 (*Bacillus halosaccharovorans*, 3.3%), OPU 172 (*Actinobacteria*, 3.0%), OPU 90 (*Sorangiineae*, 2.9%) and, OPU 22 (*Rhodobacteraceae*, 2.4%). In no case one OPU exceeded 5.1% of the total sequences (Supplementary Table S5). On the other hand, the major OPUs in BB were OPU 102 (Uncultured GR-WP33–58, 43.38%, a *Deltaproteobacteria* close to *Myxobacteria*), OPU 143 (Uncultured *Chitinophagaceae*, 12.6%), and OPU 34 (Uncultured *Limimonas*, 12.6%). The latter OPU and the OPU 109 (*Rhodopirellula*) were the unique OPUs present both in RB and BB (Supplementary Table S5).

Sequences affiliated with *Archaea* generated lower diversity yields with 26 OPUs, all them in the *Euryarchaeota* phylum (**Figure 1B**). Most of the OPUs affiliated with *Halobacteriaceae* (90.8% for RA and 100% for BA). *Methanosarcinaceae* and *Methanoregulaceae* were present only in RA with 3.9 and 5.3%, respectively. The most representative in RA sample were OPUs 204 and 205 (*Haladaptatus* sp., 52.6%), OPUs 215 and 216 (*Halopelagius* sp., 10.5%), OPUs 201–203 (*Halococcus* sp., 9.2%), OPU 226 (*Methanolinea mesophila*, 5.3%), and OPU 225 (*Methanosarcina* sp., 3.9%). While, sequences in sample BA were represented principally by OPUs 209–213 (*Halorubrum* sp., 61.2%), OPU 220 (*Haloquadratum* sp., 16.7%), OPUs 221 and 222 (*Haloarcula* sp., 3.8%), OPU 208 (*Halomarina oriensis*, 3.7%), OPU 223 (*Halonotius* sp., 3.7%), and OPU 224 (*Halobacteriaceae*, 3.7%; Supplementary Table S5).

Bacterial diversity (H′) and richness (Chao-1) indexes were higher in RB (4.5 and 221.5, respectively) than in BB (1.8 and 12, respectively; Supplementary Table S4). However, the abundances were more homogeneously distributed in RB than in BB. In accordance Dominance index for RB was the lowest in comparison with all samples (Supplementary Table S4). *Archaea* presented similar values for diversity (2.0), richness (13), and dominance (0.2) in both samples.

### Construction of Metagenomic Libraries

In order to search for genes that could confer increased salt resistance to *E. coli*, we screened two metagenomic libraries constructed in the high-copy-number vector pSKII^+^ with environmental DNA isolated from brine and from rhizosphere samples. Approximately 236,250 (brine) and 192,000 (rhizosphere) recombinant clones were obtained and the libraries were subsequently amplified as described in Experimental procedures. Fragment length polymorphism analysis of 16 random clones per library showed an average insert size of 3 kb as shown in Supplementary Table S6. Overall, ~1.2 Gb of environmental DNA was cloned within these libraries.

### Screening of the Metagenomic Libraries for NaCl Resistant Clones

**FIGURE 2:**
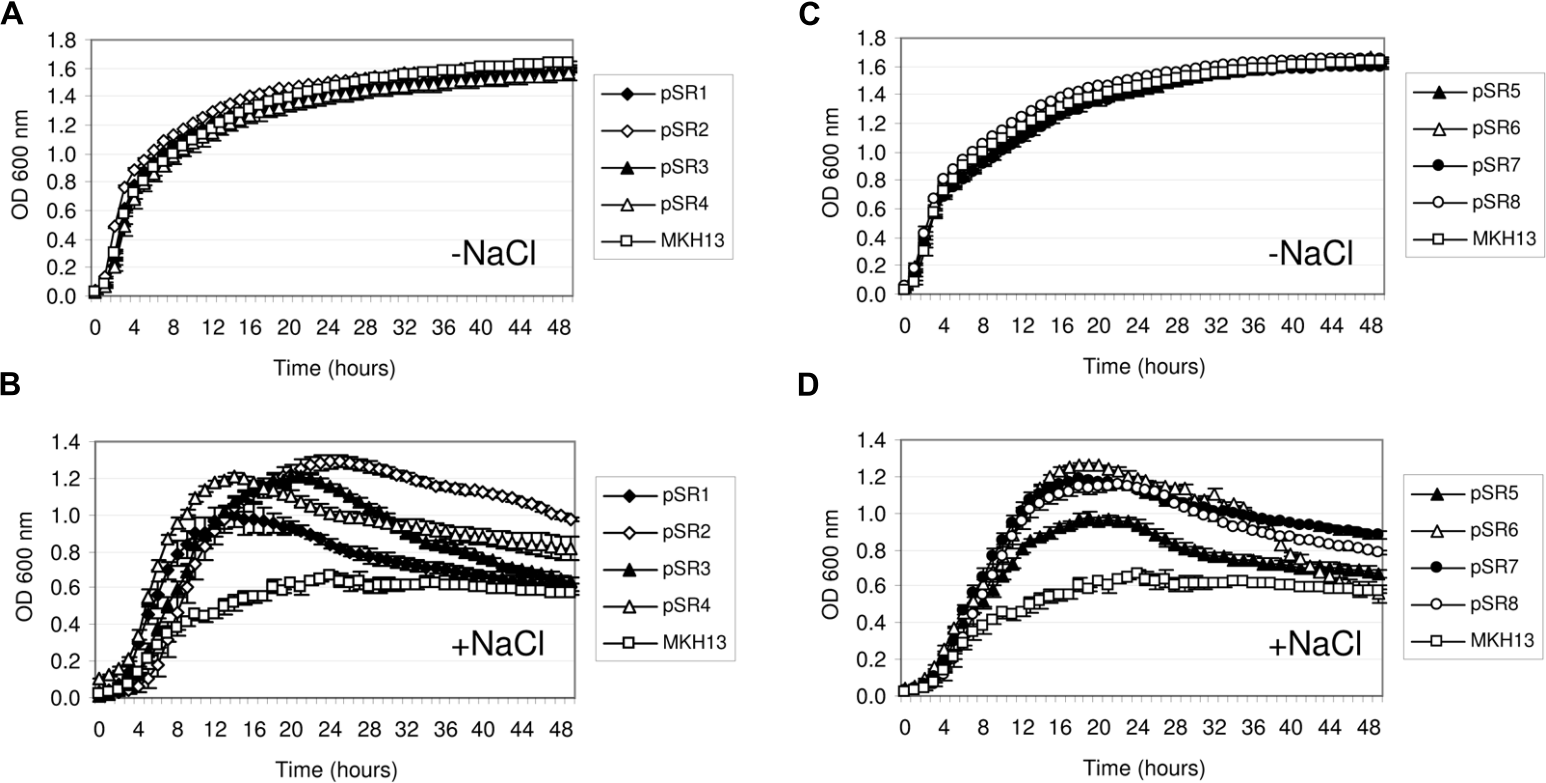
**Growth curves of *Escherichia coli* MKH13 cells carrying plasmids with salt resistance genes (pSR1-pSR8) and MKH13-pSKII^+^ in LB broth and LB broth supplemented with 3% NaCl.** Clones pSR1, pSR2, pSR3, pSR4, and MKH13-pSKII^+^ in LB broth **(A)** and LB broth supplemented with 3% NaCl **(B)**. Clones pSR5, pSR6, pSR7, pSR8, and MKH13-pSKII^+^ in LB broth **(C)** and LB broth supplemented with 3% NaCl **(D)**.

Recombinant plasmids from the two metagenomic libraries constructed in *E. coli* DH10B strain were used to transform the osmosensitive *E. coli* MKH13 strain. MKH13 is less salt-resistant than *E. coli* wild type strains, because it carries mutations in the ProP and ProU transport systems involved in the efficient uptake of the osmoprotectant proline betaine (*N*,*N*-dimethyl-L-proline; Haardt et al., 1995). One of the main problems of using *E. coli* as a host for metagenomic libraries is to obtain the appropriate expression of genes from other microorganisms. Thus, the use of the MKH13 strain could favor the selection of genes conferring salt resistance, but poorly expressed in this bacterium. As a result, a total of 101 and 12 salt resistance clones were obtained for brine and rhizosphere samples, respectively. Of these, eight clones containing genes that conferred salt resistance to the host, pSR1–3 from brine and pSR4–8 from rhizosphere (**Table 1**) were found unique in their enzymatic restriction pattern. The strain MKH13 transformed with the recombinant plasmids showed a better growth rate in LB supplemented with 3% NaCl than MKH13 cells transformed with an empty vector (**Figures 2B,D**) whereas no differences in growth rate was observed in the presence of LB medium without supplemented NaCl (**Figures 2A,C**). All the clones were also assayed in the presence of LB supplemented with 4% NaCl and an increase in the growth rate was also observed in clones pSR2, pSR4, and pSR8 (data not shown).

**Table 1:** Description of NaCl-resistant plasmids (pSR1 to pSR8) and their observed sequence similarities.

Plasmid (type of sample)	GenBank accession number no.	Size (bp)	G+C content (%)	ORF^a^	Length (aa)^b^	Closest similar protein (organism_accession number)	Domain^c^	E value	% Identity	No. of TM-helices
pSR1 (brine)	KM603475	2478	70.26	1*	171	Prolyl-tripeptidyl peptidase precursor (*Candidatus accumulibacter*); EXI70660; 322 aa	B	3,00E-72	102/161 (63%)	0
				2*	621	DNA helicase II (*Salinibacter ruber* DSM 13855); YP_003572414; 1227 aa	B	0.0	464/484 (96%)	0
pSR2 (brine)	KM603476	1509	56.20	1*	337	Hypothetical protein (*Natrinema pellirubrum*); WP_006182474; 833 aa	A	0.0	291/337 (86%)	1
				2	96	Hypothetical protein (*Halosimplex carlsbadense)*; WP_006886095; 168 aa	A	5,00E-51	80/96 (83%)	0
pSR3 (brine)	KM603477	1838	60.50	1*	392	Probable cell surface glycoprotein (*Natronomonas moolapensis* 8.8.11) YP_007488500	A	7,00E-108	212/382 (55%)	2
				2*	97	IISH7-type transposase (*Natronomonas moolapensis* 8.8.11) YP_007488498.1; 558 aa	A	8,00E-50	85/97 (88%)	0
pSR4 (rhizosphere)	KM603478	920	62.61	**1**	217	Endonuclease III (*Verrucomicrobia bacterium* DG1235); WP_008102102; 229 aa	B	1,00E-126	174/217 (80%)	0
pSR5 (rhizosphere)	KM603479	1800	64.15	1*	304	Hypothetical protein (*Monosiga brevicollis* MX1); XP_001749465; 1630 aa	E	9,00E-27	96/270 (36%)	1
				2	164	Site-specific recombinase (*Pseudomonas fuscovaginae)*; WP_029533036; 197 aa	B	4,00E-24	56/110 (51%)	0
pSR6 (rhizosphere)	KM603480	2341	49.40	1*	168	OmpA/MotB domain-containing protein (*Haliscomenobacter hydrossis* DSM 1100) YP_004451090; 1170 aa	B	5,00E-48	82/168 (49%)	0
				**2**	214	Glycerol uptake facilitator or related permease (*Methylacidiphilum infernorum* V4); YP_001939268; 209 aa	B	3,00E-38	86/202 (43%)	6
				3*	216	Hypothetical protein (*Paenibacillus daejeonensis*) WP_020618935; 465 aa (putative sulfatase)	B	1,00E-78	117/240 (49%)	0
pSR7 (rhizosphere)	KM603481	1131	54.64	1*	377	RNA helicase (*Methylophaga thiooxydans*); WP_008290720; 605 aa	B	0.0	323/383 (84%)	0
pSR8 (rhizosphere)	KM603482	426	70.66	1*	142	Pyrophosphate-energized proton pump (*Ilumatobacter coccineus* YM16–304) YP_007562997; 698 aa	B	1,00E-50	95/142 (67%)	3

*^a^ORFs involved in NaCl resistance are shown in boldface type, and asterisks indicate incomplete ORFs.*

*^b^aa, amino acids.*

*^c^A: *Archaea*, B: *Bacteria*, and E: Eukarya.*

**FIGURE 3:**
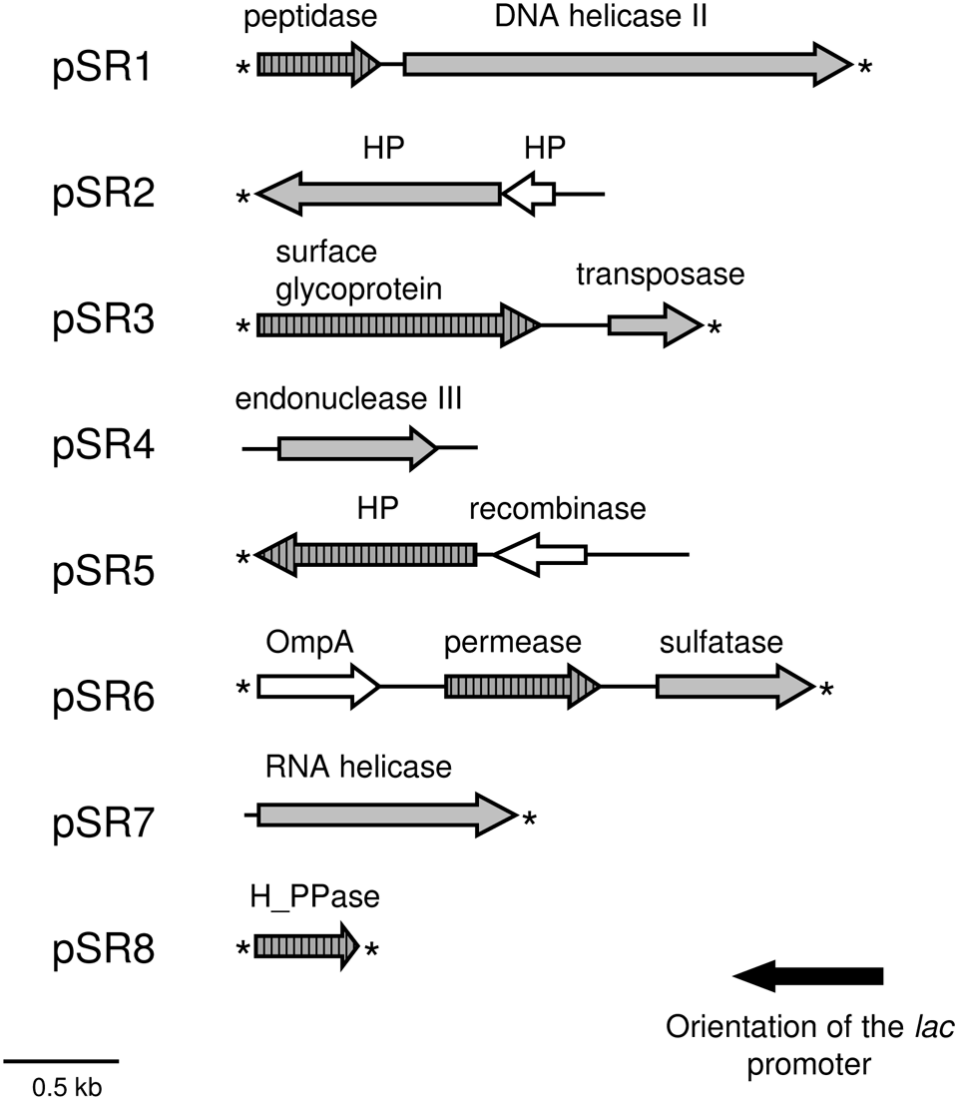
**Schematic organization of the ORFs identified in the pSR1-pSR8 plasmids.** Arrows denote the location and the transcriptional orientation of the ORFs in the different plasmids. ORFs involved in NaCl resistance are indicated by gray arrows and those whose phenotype was not resistant are shown in white arrows. The presence of predicted transmembrane helices is represented by arrows shaded with vertical bars. Asterisks indicate incomplete ORFs. HP, hypothetical protein.

A total of 14 genes were predicted using FGENESB and ORF Finder programs in the sequenced inserts from the eight plasmids (pSR1–pSR8) conferring salt resistance (**Table 1** and **Figure 3**). Sequence analyses of these environmental DNA fragments revealed the presence of one unique ORF in pSR4, pSR7 and pSR8, two ORFs in pSR1, pSR2, pSR3 and pSR5, and three ORFs in pSR6. The G+C content of these DNA fragments varied from 49.4 to 70.7% indicating their diverse phylogenetic origin. Most of the genes analyzed in this study encoded amino acid sequences similar to bacterial proteins whereas the inserts present in pSR2 and pSR3 may have been retrieved from archaeal organisms due to their similarities with members of this domain. In addition, BLASTP analyses revealed that pSR5-*orf1* may be from eukaryotic origin whereas pSR5-*orf2* was probably derived from a bacterium related to the *Pseudomonas* genus. This result suggests that pSR5 may be a chimeric clone or that this clone may be derived from a fragment of a mobile element. Alternatively, pSR5-*orf1* may be just an uncommon bacterial gene with the eukaryotic sequence being the closest gene sequenced. BLASTP as well as the protein family domains (Pfam) databases were used to functionally categorize the genes retrieved and showed that pSR1-*orf2* and pSR4-*orf1* encoded proteins related to DNA repair processes such as a DNA helicase II and an endonuclease III, respectively (**Table 1** and Supplementary Table S7). It is also interesting to note that genes related to structural dynamics of nucleic acids were also retrieved, including a IISH7-type transposase encoded by pSR3-*orf2*, a putative site-specific recombinase encoded by pSR5-*orf2* and a putative RNA helicase, particularly a DEAD-box helicase encoded by pSR7-*orf1* (**Table 1**). The deduced amino acid sequence of pSR7-*orf1* contained the five conserved sequence motifs found in members of the DEAD-box helicase family: II or Walker B (VLDEADEM; positions 10–17), III (SAT; positions 43–45), IV (IIFVRT; positions 105–110); V (LVATDVAARGLD; positions 155–166) and VI (YVHRIGRTGRAG; positions 185–196). Putative proteins encoded by pSR3-*orf1*, pSR6-*orf2*, and pSR8-*orf1* were similar to a cell surface glycoprotein, a permease related to glycerol uptake and a proton pump, respectively. These may be related to either transport mechanisms or to membrane components, in agreement with the presence of transmembrane segments predicted in their amino acid sequences (**Table 1**). The protein encoded by pSR6-*orf3* showed homology with choline-sulfatases from *Vibrio* sp., *Cyclobacterium qasimii* and *Clostridiales*. Also, it contained the motif SDHGEFL (positions 71–77), which is highly similar to a peptide signature apparently specific to choline sulfatases SDHGDML (Cregut et al., 2014).

In addition, hypothetical proteins were also found, such as those encoded by pSR2-*orf1*, pSR2-*orf2*, pSR5-*orf1*, and pSR6-*orf3*. In the case of pSR5-*orf1*, Pfam analysis showed that the encoded protein contained a VWA (von Willebrand factor type A) domain present in some eukaryotes (Supplementary Table S7).

### Identification of Genes Conferring NaCl Resistance

The recombinant plasmids pSR4, pSR7, and pSR8 contained a single ORF each, encoding an endonuclease III, a RNA helicase and a proton pump, respectively, which are responsible for the NaCl resistance phenotype (**Table 1**, **Figures 2B,D**). Five recombinant plasmids contained more than one ORF (pSR1, pSR2, pSR3, pSR5, and pSR6) as shown in **Table 1** and **Figure 3**. The DNA insert of pSR1 contains two ORFs: *orf1* encoding a peptidase S9 and *orf2* encoding a DNA helicase II. Clones harboring each one of these ORFs were NaCl resistant since an increase in the growth rate was observed compared to the growth of MKH13-pSKII^+^cells, and even slightly more pronounced than that of the original clone (Supplementary Figure S1). In the case of the DNA insert from pSR2, two ORFs were identified, both encoding hypothetical proteins. pSR2-*orf1* clearly conferred resistance to NaCl whereas the slight resistance observed in the growth of pSR2-*orf2* (Supplementary Figure S2B) may be explained by its limited growth in LB not supplemented with NaCl (Supplementary Figure S2A). The sequence of the DNA insert of pSR3 plasmid revealed that it contained two ORFs, *orf1* encoded a probable cell surface glycoprotein whereas *orf2* encoded a IISH7-type transposase. These two genes were both involved in the NaCl resistance observed in the original clone as shown in Supplementary Figure S3. In the case of the DNA sequence of pSR5 two ORFs were identified and whose amino acid sequences were similar to a hypothetical protein (*orf1*) and to a recombinase (*orf2*). The increased growth rates observed for these clones revealed that pSR5-*orf1* provided NaCl resistance when compared with that of MKH13-pSKII^+^, and its growth rate was similar to that of the original clone although slightly delayed (Supplementary Figure S4), whereas the growth rate of the clone harboring pSR5-*orf2* was reduced when compared with that of the control strain in the LB medium supplemented with NaCl (Supplementary Figure S4B). Three ORFs were found in the DNA insert of pSR6, encoding a protein similar to an OmpA (*orf1*), a permease involved in glycerol uptake (*orf2*) and a putative permease (*orf3*). Clones containing *orf2* and *orf3*, but not *orf1*, exhibited higher growth rates than that observed in the control (MKH13 pSKII^+^) in LB medium supplemented with NaCl, indicating that *orf2* and *orf3* may be responsible for the NaCl resistance observed in the original clone (Supplementary Figure S5).

### Assessment of Salt Resistance in the *E. coli* Homologs of Environmental Genes

**FIGURE 4:**
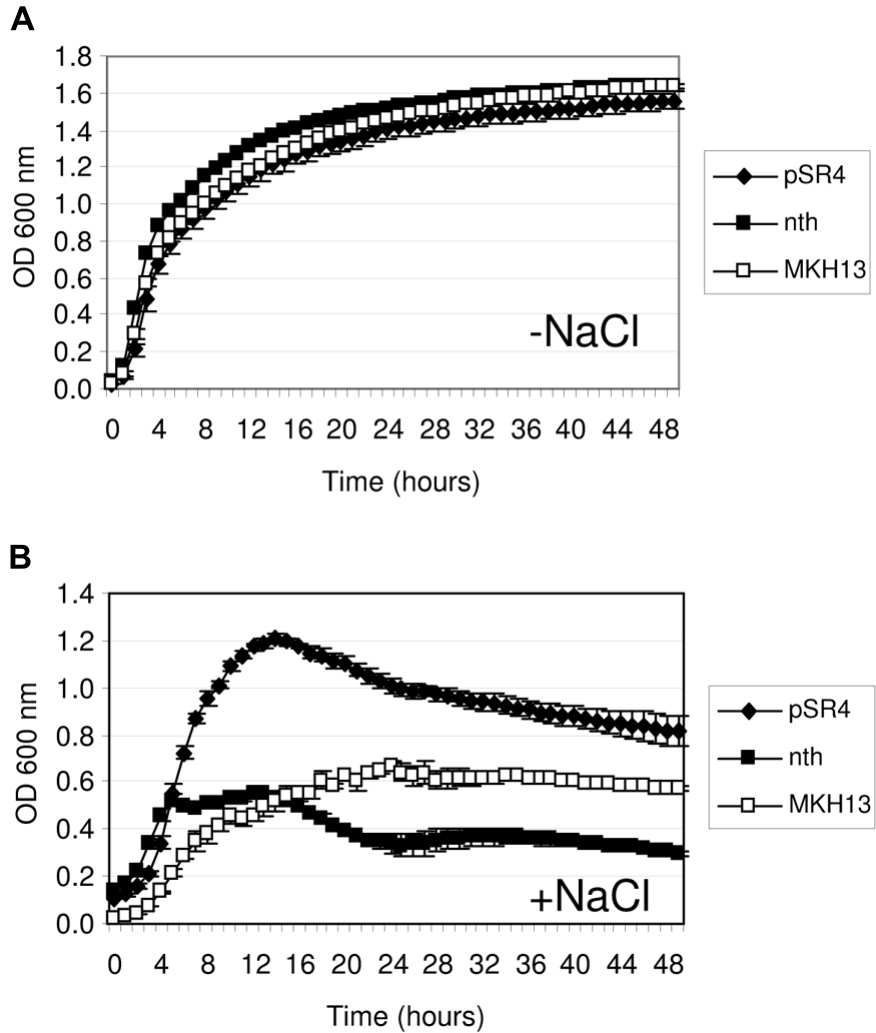
**Growth curve of *E. coli* MKH13 cells carrying pSR4, the *E. coli nth* gene, and MKH13-pSKII^+^ in LB broth **(A)** and LB broth supplemented with 3% NaCl (B)**.

**FIGURE 5:**
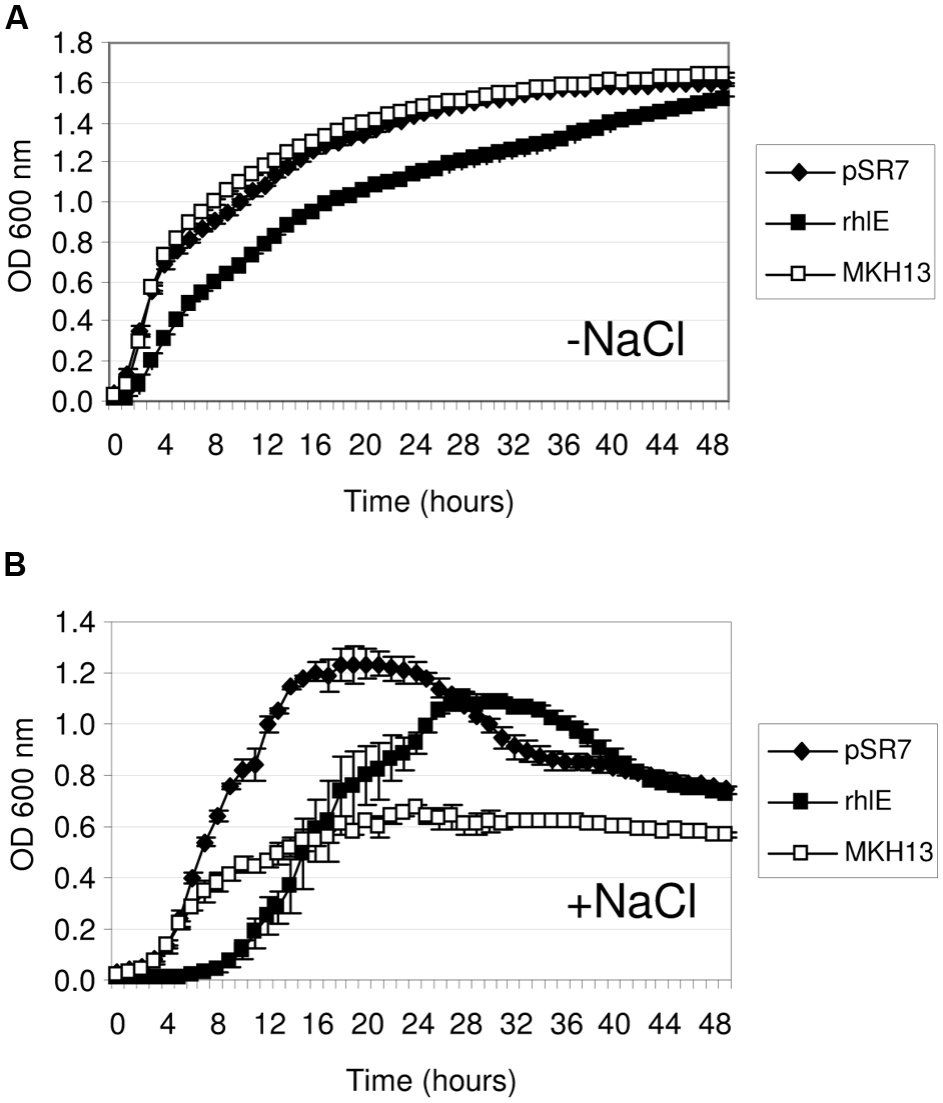
**Growth curve of *E. coli* MKH13 cells carrying pSR7, the *E. coli rhlE* gene and MKH13-pSKII^+^ in LB broth **(A)** and LB broth supplemented with 3% NaCl (B)**.

The discovery of salt-resistance genes related to nucleic acid metabolism has been an interesting finding in this work. Thus, to explore the specificity of these environmental genes in the resistance phenotype, their *E. coli* homologs were cloned and tested for growth in the presence of NaCl. The proteins encoded by pSR4-*orf1* and pSR7-*orf1* were similar to the endonuclease III (Nth, 38.53% identity; 49.54% similarity) and the DEAD-box RNA helicase (RhlE, 31.94% identity; 46.86% similarity) of *E. coli*, respectively. These genes were PCR amplified using genomic DNA from MKH13 cells, digested with either XhoI or HindIII and XbaI and ligated into pSKII^+^ digested with the same restriction enzymes. The growth on LB supplemented with 3% NaCl of the clones harboring the environmental genes, their *E. coli* homologs and the empty plasmid were compared. As a result, the growth rates of the strain carrying the *nth* gene of *E. coli* and the control strain (MKH13 pSKII^+^) were similar in contrast with the increased growth rate observed for the clone pSR4 (**Figure 4**), indicating that the environmental endonuclease III but not its *E. coli* homolog specifically conferred salt resistance. The growth of the clone carrying the pSR7 plasmid, which encoded a protein similar to a DEAD-box RNA helicase, and the clone containing the *rhlE* gene of *E. coli* were also compared. As a result, we observed a reduced growth rate of the *rhlE* clone in the presence of LB alone and a prolonged lag phase in the presence of NaCl (**Figure 5**). These results suggest that the RNA helicase of environmental origin may provide a faster adaptation to the presence of NaCl in LB medium than its *E. coli* homolog.

### Expression of Salt Resistance Genes in *Bacillus subtilis*

**FIGURE 6:**
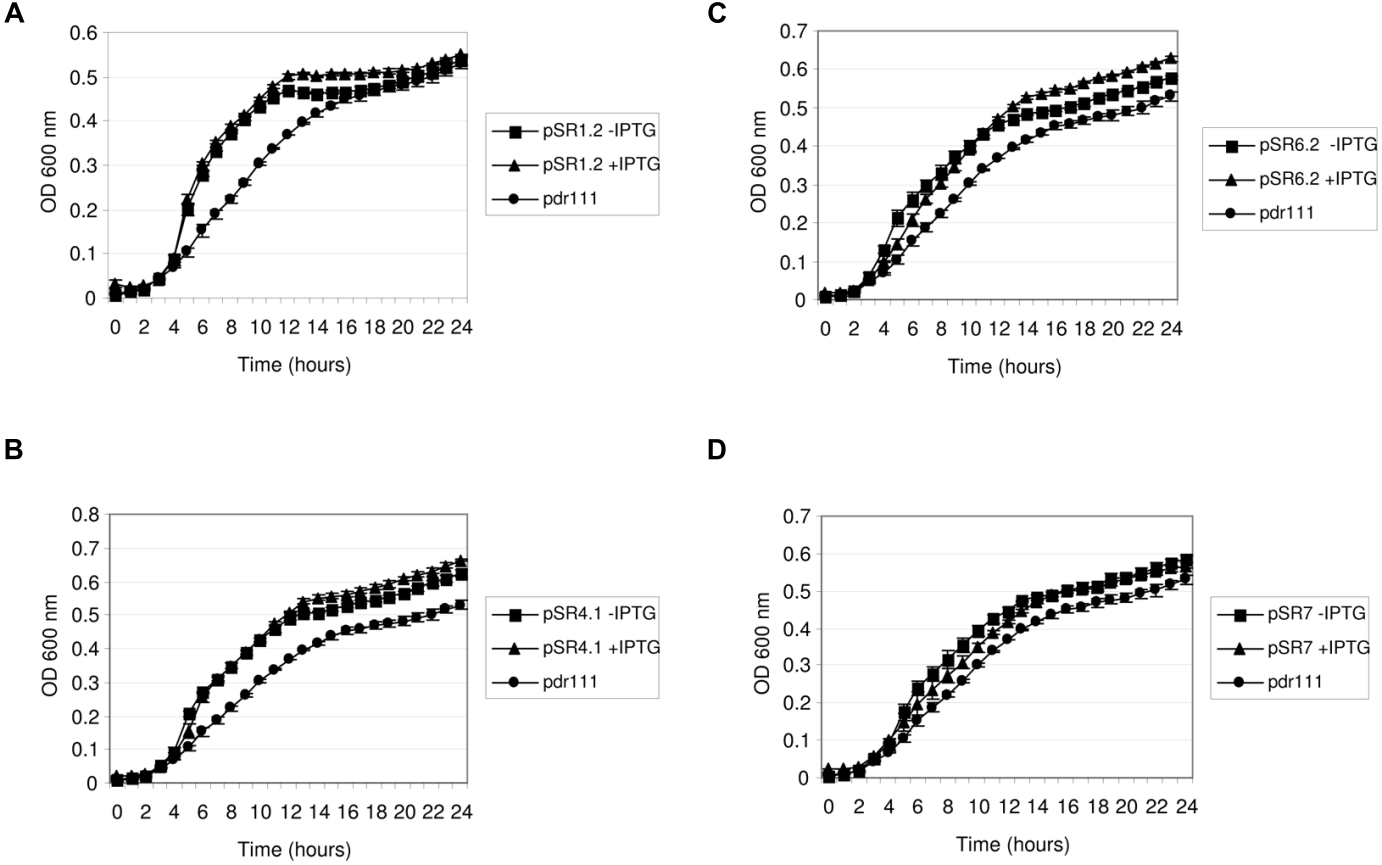
**Growth of *Bacillus subtilis* clones in NaCl.** *B. subtilis* clones pSR1-*orf2* **(A)**, pSR4-*orf1* **(B)**, pSR6-*orf2* **(C)** and pSR7-*orf1* **(D)** were grown in LB broth supplemented with 6% NaCl in the presence and in the absence of 1mM IPTG. *B. subtilis* strain PY79 with the empty plasmid pdr111inserted in the chromosome was used as negative control.

In order to investigate the expression of some of the retrieved environmental genes involved in salt resistance in other hosts than *E. coli*, four of the identified genes were transferred to the model organism *B. subtilis* (PY79 strain). This bacterium was chosen as a representative of Gram-positive bacteria because it is suitable for genetic manipulation (Earl et al., 2008). PY79 strain exhibited increased resistance to NaCl than *E. coli* MKH13, thus salt concentration was adjusted to 6% in the growth experiments. The genes selected to be expressed in *B. subtilis* were those related to metabolism of nucleic acids (pSR1-*orf2*, pSR4-*orf1*, and pSR7-*orf1*) and also one encoding for a protein similar to a permease (pSR6-*orf2*). These four genes were subcloned into pdr111 vector, under an inducible IPTG promoter, the hyper-SPANK promoter. The resulting constructions were inserted at the *amyE* locus in the *B. subtilis* chromosome. In the growth experiments, bacteria carrying the empty vector inserted in the chromosome were used as negative control. Interestingly, *B. subtilis* transformed with these genes and grown either in the presence or in the absence of IPTG exhibited an increased growth rate in comparison with the negative control, as shown in **Figure 6**. These results indicated that some basal level of expression is occurring when *B. subtillis* was transformed with these environmental genes. From these, all the clones but pSR7-*orf1* showed a slight higher growth rate in the presence of salt in the medium when IPTG was supplemented than those without it, indicating that these genes were induced by IPTG, and properly expressed by *B. subtilis*, conferring resistance to NaCl.

### Determination of Cellular Na^+^ Content

**FIGURE 7:**
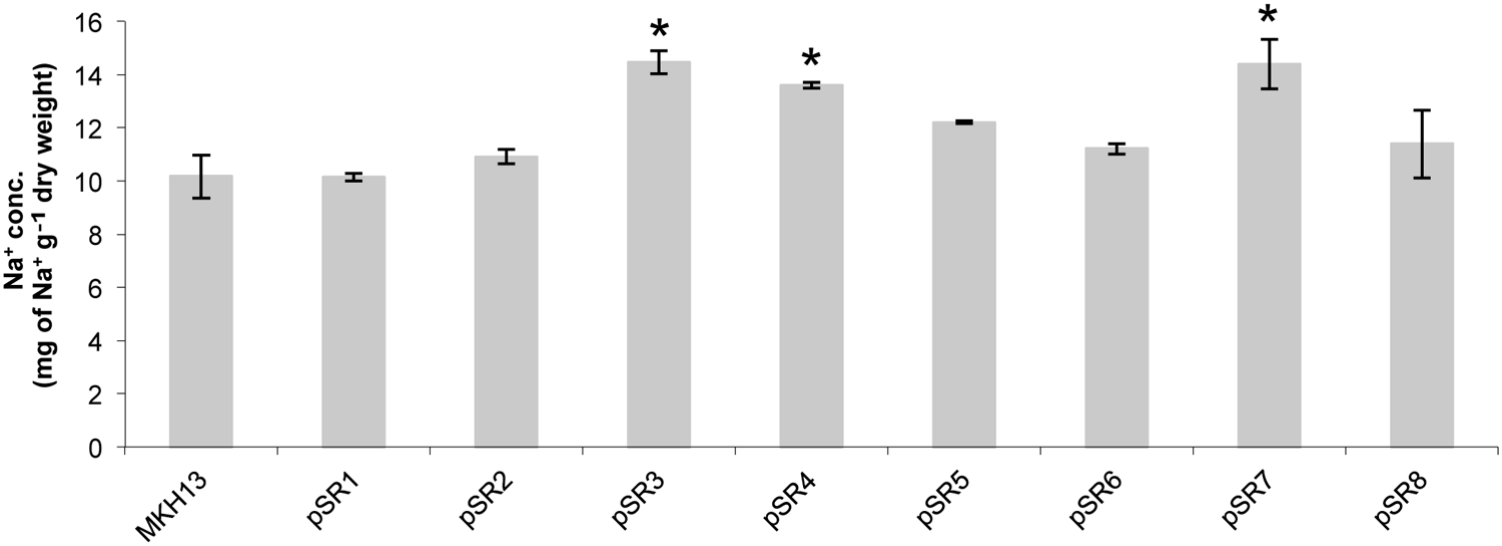
**Test for cellular content of Na^+^ ion in *E. coli* clones pSR1 to pSR8 and MKH13-pSKII^+^ after 1 h of growth with 6% NaCl.** Values are the averages of two independent ICP-MS measurements. Error bars indicate standard deviation. An asterisk indicates significantly different from control cells as determined by one-way ANOVA followed by Tukey’s test (*p* < 0.05).

To assess the extent by which clones pSR1 to pSR8 can accumulate Na^+^ ions, the cellular concentration of this element was measured by ICP-MS after 1 h of growing bacterial cells with 6% NaCl (**Figure 7**). From the quantification of Na^+^, resistant clones were grouped into two categories according to whether these clones can accumulate more or less sodium. The first group consisted of clones which accumulated more sodium than the control (pSR3, pSR4, and pSR7). This included clones involved in DNA repair such as the endonuclease III encoded by pSR4-*orf1*. The second group showed the same sodium concentration in the cell compared to the control cells (pSR1, pSR2, pSR5, pSR6, and pSR8). This included clones carrying genes related to the modification of DNA such as the DNA helicase II (pSR1-*orf2)* or of unknown function (pSR2-*orf1*). It also included clones with genes that may be involved in osmotic equilibrium such as pSR6 with two genes, pSR1-*orf2* and pSR6-*orf3*, encoding a glycerol permease and a putative sulfatase, respectively and pSR8, with one gene, pSR8-*orf1*, encoding for a proton pump.

**FIGURE 8:**
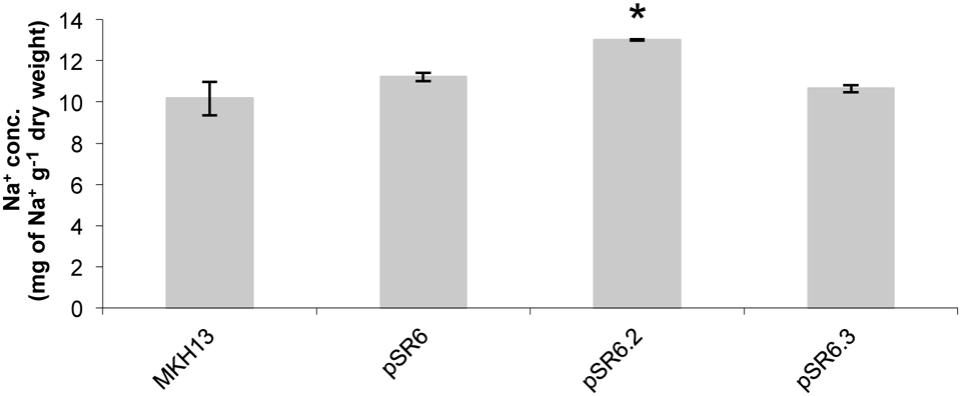
**Test for cellular content of Na^+^ ion in *E. coli* clones pSR6, pSR6-*orf2*, pSR6-*orf3*, and MKH13-pSKII^+^ after 1 h of growth with 6% NaCl.** Values are the averages of two independent ICP-MS measurements. Error bars indicate standard deviation. An asterisk indicates significantly different from pSR6, pSR6-*orf3* and control cells as determined by one-way ANOVA followed by Tukey’s test (*p* < 0.05).

Further quantification of the cellular content of Na^+^ ions determined by ICP-MS on the pSR6 clone revealed that the recombinant plasmid encoding only the putative permease, pSR6-*orf2*, accumulated significantly more sodium than the original and pSR6-*orf3* clones and also more than MKH13 cells (**Figure 8**).

## Discussion

Functional metagenomics allows access to the potential genetic diversity of both cultured and uncultured bacteria present in a particular environment (Handelsman, 2004). Therefore, this approach was used in this study to decipher the molecular mechanisms that may contribute to the overall cellular resistance and by which microbial communities adapt to high salt content. This has been employed in diverse studies aimed to elucidate the mechanisms of adaptation of microbial consortia to a number of extreme conditions such as high nickel and arsenic content, and acidic pH from the acid mine drainage environment of Rio Tinto (Mirete et al., 2007; González-Pastor and Mirete, 2010; Guazzaroni et al., 2013; Morgante et al., 2014). Although functional metagenomics has been applied to screen for genes related to salt resistance in environmental samples from the human gut microbiome (Culligan et al., 2012, 2013), and also from a freshwater pond (Kapardar et al., 2010), to the best of our knowledge this is the first study to report novel salt resistance determinants from microorganisms of a hypersaline environment by using functional screening of metagenomic libraries.

The two samples from which the metagenomes originated exhibited a microbial composition in accordance with the kind of sample (soil or brine) and high salinities. The rhizosphere was very diverse in its bacterial composition with 187 distinct OPUs in accordance with the known complexity of the system (Philippot et al., 2013). The relative abundances of the representatives of each lineage were well-balanced and none exceeded the 5.1% of the total diversity. The composition of the main taxonomic groups were *Alpha*- and *Gammaproteobacteria* and especially deltaproteobacterial which are close relatives to *Myxobacteria*, together with *Actinobacteria, Firmicutes, Bacteroidetes*, and *Gemmatimonadetes* are known to be common inhabitants of rhizosphere soils (Philippot et al., 2013). It is worth noting the relative high abundances of organisms related to *A. maritima*, a *Chloroflexi* representative known as an iron and nitrate reducer (Kawaichi et al., 2013), and *B. halosaccharovorans*, a moderately halophilic *Firmicutes*, both in accordance with the saline conditions of the environment (Mehrshad et al., 2013). The archaeal composition was less complex with only representatives of the *Halobacteriaceae* family in accordance with the high salinity concentrations (Oren, 2008), and representatives of the Rice Cluster I methanogens (*Methanosarcinales* and *Methanomicrobiales*; Conrad et al., 2006) also common in soils and widely distributed. The most remarkable observations were the high abundance (over 50% of the total archaeal diversity) of a close relative of the halobacterial genus *Haladaptatus*, originally isolated from low-salt and sulfide rich environments (Savage et al., 2007); and the methanogenic species *M. mesophila* initially described in rice field soil (Sakai et al., 2012), and member of the Rice Cluster I (Conrad et al., 2006). Altogether the results on the community structure of this soil agree with the fact that the anaerobic hypersaline sediments below the brine crystallizers may be a source of methane and sulfide (López-López et al., 2010), and these may influence (by diffusion of ions and migration of microorganisms) the surrounding soils from which the plants were sampled.

The microbial composition of the salt brines was remarkable. The archaeal community was only constituted by members of *Halobacteriaceae* and with the genera *Haloquadratum, Halorubrum*, and *Haloarcula* as the most abundant. This structure was in accordance with the known microbiota in brines (Oren, 2008). However, the bacterial composition was remarkably different from what was expected. In general *Salinibacter* representatives have been found to be the major bacterial fraction in brines, in proportions that range from 5 to 30% (Antón et al., 2008). However, despite sequences of this lineage being found in the brines studied here, these constituted a minority (about 5% of the total bacterial diversity). The most conspicuous observation was the detection of three major groups of bacteria not previously observed as major components with ecological relevance in hypersaline habitats. The most represented bacterial lineage affiliated with representatives of the uncultured myxobacterial clade GR-WP33–58. Sequences of this deltaproteobacterial lineage were first detected in deep-sea Antarctic samples (Moreira et al., 2006). However, since its initial detection, similar sequences were retrieved mostly in marine samples (according to the identifiers in the entries from the NCBI). Some sequences of this clade had also been retrieved from hypersaline microbial mats (Harris et al., 2013) and saline soils (Castro-Silva et al., 2013), pointing to that its presence in brines may not be anomalous. The second most relevant proteobacterial group detected, and also in higher sequence abundances than *Salinibacter* were relatives of *Limimonas* (Amoozegar et al., 2013), an extremely halophilic member of *Rhodospirillaceae.* Finally, a third relevant group affiliated with relatives of the *Chitinophagaceae* lineage within *Bacteroidetes.* Similar sequences were detected in the hypersaline Lake Tyrrel in Australia (Podell et al., 2013). Despite the sequences retrieved for the bacterial domain being in accordance with the hypersaline nature of the sample, the lower occurrence of *Salinibacter*, and the prevalence of representatives from the uncultured GR-WP33–58 clade need further investigation as such community structure has not been observed before.

The construction of metagenomic libraries and their subsequent functional screening to search for novel salt resistance genes was considered in this study taking into account the microbial diversity observed in the brine and rhizosphere samples. It is worth to note that the genes identified here and those found in the natural host may not be involved in a similar degree of salt tolerance. In general, a correlation was observed between the putative phylogenetic affiliation of the environmental DNA fragments present in the positive clones and the sample origin (brine or rhizosphere). For example ORFs identified in clones derived from the brine sample (pSR1–pSR3) were similar to those from organisms detected in brine samples such as members of *Salinibacter* and *Halobacteriaceae* whereas ORFs from clones derived from the rhizospheric soil (pSR4–pSR8) were assigned to microorganisms found in this sample including representatives of *Gammaproteobacteria*, *Firmicutes*, *Verrucomicrobia*, *Bacteroidetes*, and *Actinobacteria*.

In microorganisms, a well-known response to salt stress is the increase in concentration in the cytoplasm of compatible solutes such as glycerol and glycine betaine, in response to an elevated osmolarity in the surrounding medium. The synthesis of these solutes is often energetically less favorable than the uptake from the external environment and thus the accumulation of compatible solutes can inhibit endogenous synthesis (Sleator and Hill, 2001). The finding of pSR6-*orf2*, which encoded a putative glycerol permease, and conferred NaCl resistance not only in *E. coli* MKH13 but also in *B. subtilis*, illustrates the presence of this strategy within the rhizosphere bacterial community. Also, pSR6-*orf3* encoded a putative choline sulfatase, which was responsible for the resistance phenotype observed when it was cloned independently. Choline sulfatases encoded by *betC* genes are necessary to convert choline sulfate into choline and are found in several microorganisms present in rhizospheric environments including *Sinorhizobium meliloti* (Østerås et al., 1998). Although the *betC* gene is absent within the *E. coli* genome, we can assume that the presence of a gene encoding a choline sulfatase may favor the synthesis of glycine betaine from choline since in *E. coli* cells this last conversion can be carried out through two oxidations steps catalyzed by a choline dehydrogenase (BetA) and a glycine betaine aldehyde dehydrogenase (BetB; Østerås et al., 1998; Sleator and Hill, 2001). It is interesting to note that only the clone carrying pSR6-*orf2* accumulated more Na^+^ than the control, the original clone pSR6 and pSR6-*orf3*.

In addition, an ORF from pSR8 encoding a proton pumping membrane-bound pyrophosphatase (H^+^-PPase) was identified in this study. These proteins have been found in all three domains of life and can confer resistance to cells against diverse abiotic stress such as cold, drought, NaCl and metal cations, probably because the enzyme generates a membrane potential by using PPi (Yoon et al., 2013; Tsai et al., 2014). Membrane-bound pyrophosphatases can require Na^+^ for their activity and they can also catalyze the transport of Na^+^ outside the cell, as it has been demonstrated in the archaeal PPase from the mesophile *Methanosarcina mazei* and in two bacterial PPases from the hyperthermophile *Thermotoga maritima* and the moderate thermophile *Moorella thermoacetica* (Malinen et al., 2007). More recently, an integral membrane pyrophosphatase subfamily has been described in diverse bacterial species which has the ability to transport both Na^+^ and H^+^ outside bacterial cells and which may have evolved from Na-PPases (Luoto et al., 2013). Thus, the membrane-bound pyrophosphatase encoded by pSR8-*orf1*, coupled with Na^+^/H^+^ antiporters present in *E. coli*, may be playing an important role in the adaptation of bacterial cells to increased salt content (Baykov et al., 2013).

A relevant finding derived from this study is the identification of salt resistance genes related to DNA repair and to structural dynamics of nucleic acids. Examples of these genes are pSR1-*orf2* and pSR7-*orf1*, which encoded a DNA and a DEAD-box RNA helicase, respectively. These genes were also responsible for the NaCl resistance phenotype observed in *B. subtilis*. Interestingly, the environmental RNA helicase encoded by pSR7 showed better adaptation to NaCl than that cloned from *E. coli*. DNA helicases are involved in unwinding double strand DNA and thus play key roles in cellular processes such as recombination, replication, transcription and repair processes whereas RNA helicases are capable of unwinding RNA duplexes and thus participate in ribosome biogenesis, transcription, translation initiation and RNA degradation (Tanner and Linder, 2001; Delagoutte and von Hippel, 2002; Kaberdin and Bläsi, 2013). In bacteria, DEAD-box RNA helicases involved in cold and oxidative stress response have been reported in the cyanobacterium *Anabaena* sp. (Yu and Owttrim, 2000) and in *Clostridium perfringens* (Briolat and Reysset, 2002), respectively. Also, upregulation of both RNA and DNA helicases transcript levels has been observed when *Desulfovibrio vulgaris* was exposed to elevated sodium chloride concentration (Mukhopadhyay et al., 2006). The role played by these helicases may be similar to that observed in other enzymes involved in the molecular conformation of nucleic acids. In plants, these proteins have been shown to be also related to salt stress. For example, the DEAD-box DNA/RNA helicase from pea overexpressed in tobacco conferred increased salt resistance (Sanan-Mishra et al., 2005) and DEAD-box RNA helicases are induced under elevated salt conditions in *Hordeum vulgare* (Nakamura et al., 2004) and in the halophyte *Apocynum venetum* (Liu et al., 2008). In our study, the cells carrying the DEAD-box RNA helicase encoded by pSR7-*orf1* showed more accumulation of Na^+^ ions than the control, which was also reported in the leaves of transgenic tobacco plants overexpressing the DEAD-box helicase (Sanan-Mishra et al., 2005). Thus, this protein may be linked to a more specific response to salt stress that may allow the accumulation of Na^+^ ions inside the cell. This will be the basis for future studies to clarify the precise molecular mechanism of salt resistance conferred by the DEAD box DNA/RNA helicases.

A resistance phenotype to NaCl was observed in clone pSR4, which encoded a protein similar to an endonuclease III. In *E. coli* this protein is encoded by the *nth* gene and displays DNA glycosylase activity involved in base-excision repair as a cellular defense against a variety of DNA damages caused by desiccation and UV irradiation (Kish and DiRuggiero, 2012). The enzymatic activity of Nth is specific for the repair of oxidized bases in DNA, particularly pyrimidines substrates such as thymine glycol, 5-hydroxycytosine and 5-hydroxyuracil (Dizdaroglu, 2005). Repair of oxidized DNA bases after exposure to elevated doses of gamma radiation has been reported in the extremely halophilic archaeon *Halobacterium salinarum* (Kish et al., 2009) whose genome contains diverse homologs of DNA glycosylases including *nth* homologs (Dassarma et al., 2001). The endonuclease III identified in this study, which also conferred salt resistance in *B. subtilis* (**Figure 6**), was similar to the *E. coli* Nth, however, the latter did not confer salt resistance (**Figure 4**). Although, to the best of our knowledge, the effect of high salt concentrations on DNA modifications *in vivo* has not been described before, our results suggest the possibility of a specific role in repairing DNA lesions produced by NaCl in both *E. coli* and *B. subtilis* cells. Also, in the human gut environment, two genes encoding MazG were found to be involved in salt tolerance, and it was suggested that this protein may play a role in the removal of abnormal nucleotides from nascent DNA strands (Culligan et al., 2012). Diverse DNA repair pathways have been identified to withstand diverse environmental stress associated to hypersaline environments such as ionizing radiation (IR) or desiccation in halophiles (Kish and DiRuggiero, 2012) and also in the rhizosphere-associated bacterium, *Sinorhizobium meliloti* (Humann et al., 2009), which is in agreement with the rhizosphere origin of pSR4-*orf1*.

## Conclusion

The two different samples from a hypersaline environment (i.e., brine and rhizosphere) studied in this work exhibited a microbial composition that was in agreement with their saline nature. The rhizospheric soil showed a balanced community structure comparable with other such samples. The brine community structure was in agreement with what was expected for the archaeal counterpart, but not for the bacterial composition. Conspicuously, the bacterial diversity was dominated by three lineages never reported as major components of hypersaline habitats, and the expected major key player *Salinibacter* was in a noticeable minority. The use of functional metagenomics allowed the identification of diverse genes conferring salt resistance to *E. coli* and encoding for: (i) well-known proteins involved in osmoadaptation such as a glycerol permease and a proton pump, (ii) proteins related to repair, replication and transcription of nucleic acids such as RNA and DNA helicases and an endonuclease III, and (iii) hypothetical proteins of unknown function. It is worth noting that the environmental endonuclease III and the hypothetical proteins identified here may represent novel mechanisms of osmoadaptation. The link between DNA repair enzymes and stress processes involved in cellular dehydration such as desiccation and UV radiation have been previously described in *Deinococcus radiodurans* (Mattimore and Battista, 1996; Kish and DiRuggiero, 2012). To our knowledge this is the first report to identify a specific DNA repair gene from a moderate-salinity rhizosphere associated with a hypersaline environment which can provide salt resistance to *E. coli*. Further analysis of these genes will be necessary to elucidate their precise mechanism of action.

## Conflict of Interest Statement

The authors declare that the research was conducted in the absence of any commercial or financial relationships that could be construed as a potential conflict of interest.

## Footnotes

1 http://www.ncbi.nlm.nih.gov/gorf/gorf.html

2 http://pfam.xfam.org/

3 http://www.ch.embnet.org/software/TMPRED_form.html.
