# Supplementary material for: Salt resistance genes revealed by functional metagenomics from brines and moderate-salinity rhizosphere within a hypersaline environment
Source: Front Microbiol. 2015 Oct 13;6:1121. doi: 10.3389/fmicb.2015.01121 (PMC4602150; doi:10.3389/fmicb.2015.01121)
Supplement: Supplementary file 1 [file Presentation_1.PDF]

frontiers

Supplementary Material

Functional metagenomics study of salt resistance genes within a hypersaline environment

Salvador Mirete[1], Merit R. Mora-Ruiz[2], María Lamprecht-Grandío[1], Carolina G. de Figueras[1], Ramón Rosselló-Móra[2] and José Eduardo González-Pastor[1*]

[1] Laboratory of Molecular Adaptation, Department of Molecular Evolution. Centro de Astrobiología (CSIC-INTA), Torrejón de Ardoz , Madrid, Spain

[2] Marine Microbiology Group, Department of Ecology and Marine Resources, Mediterranean Institute for Advanced Studies (IMEDEA, CSIC-UIB), Esporles, Spain

*** Correspondence:** José Eduardo González-Pastor, Laboratory of Molecular Adaptation, Department of Molecular Evolution. Centro de Astrobiología (CSIC-INTA), Carretera de Ajalvir km 4, Torrejón de Ardoz, 28850, Madrid, Spain
gonzalezpje@cab.inta-csic.es

1. Supplementary Figures and Tables

1.1. Supplementary Tables

Supplementary Table 1. Primer pair sequences used for 454-pyrosequencing.

**First amplification**				
	Sequence (5’ → 3’)			
GM3 (B)*	AGAGTTTGATCMTGGC			
630 (B)	CADAAAGGAGGTGATCC			
21F (A)**	TTCCGGTTGATCCTGCCGGA			
1492R (A)	TACGGYTACCTTGTTACG			
**Second amplification**				
	Sequence (5’ → 3’)			
	Adaptor	Key	MID	Primer
GM3-PS	CCTATCCCCTGTGTGCCT TGGCAGTC	TCAG	-	AGAGTTTGATCMT GGC
21F-PS	CCTATCCCCTGTGTGCCT TGGCAGTC	TCAG	-	TTCCGGTTGATCCT GCCGGA
907-PS	CCTATCCCCTGTGTGCCT TGGCAGTC	TCAG	-	CCGTCAATTCMTT TGAGTT
**Sample**				
RB			AGCACTGTAG	
BB			ACGACTGCGT	
RA			ACGCTCGACA	
SA			AGACGCACTC	

**Bacteria* and ***Archaea* primers

Supplementary Table 2. Primers used for subcloning in plasmid pSKII+ in *E. coli* **(A)** the different environmental ORFs and **(B)** the *E. coli* homologs *nth* and *rhlE*.

(A) Primers used for subcloning environmental ORFs

Primer name	Sequence (5´-3´)	Restriction enzyme	PCR length product (bp)
pSR1-ORF1F	*CAGGTCTCGAGCGATAAGCTTGATATCGAATTC	XhoI	579
pSR1-ORF1R	CAGGTTCTAGAGTGGAGAGCGAGAGAATG	XbaI	
pSR1-ORF2F	CAGGTCTCGAGCGATCACTACCTGAAGGAC	XhoI	2077
pSR1-ORF2R	*CAGGTTCTAGACAATTAACCCTCACTAAAGGG	XbaI	
pSR2-ORF1F	*CAGGTCTCGAGCGATAAGCTTGATATCGAATTC	XhoI	1169
pSR2-ORF1R	CAGGTTCTAGAGCATATACTGACTGACGATG	XbaI	
pSR2-ORF2F	CAGGTCTCGAGGTATGCTACTTTCCACTGAC	XhoI	485
pSR2-ORF2R	*CAGGTTCTAGACAATTAACCCTCACTAAAGGG	XbaI	
pSR3-ORF1F	*CAGGTCTCGAGCGATAAGCTTGATATCGAATTC	XhoI	1250
pSR3-ORF1R	CAGGTTCTAGAGTCGATACCACCCTCGTTG	XbaI	
pSR3-ORF2F	CAGGTCTCGAGGACTGTCTTCGGTCTAGTC	XhoI	481
pSR3-ORF2R	*CAGGTTCTAGACAATTAACCCTCACTAAAGGG	XbaI	
pSR5-ORF1F	*CAGGTCTCGAGCGATAAGCTTGATATCGAATTC	XhoI	1111
pSR5-ORF1R	CAGGTTCTAGACTACTCTCGCATCTGTACAC	XbaI	
pSR5-ORF2F	CAGGTAAGCTTCTGTCGCTACCGAAGAGG	HindIII	668
pSR5-ORF2R	CAGGTTCTAGAGTAGCTCAGTCGGTAGAG	XbaI	
pSR6-ORF1F	*CAGGTCTCGAGCGATAAGCTTGATATCGAATTC	XhoI	603
pSR6-ORF1R	CAGGTTCTAGAGAATGCATGAATGCCATAATG	XbaI	
pSR6-ORF2F	CAGGTCTCGAGCATTATGGCATTCATGCATTC	XhoI	952
pSR6-ORF2R	CAGGTTCTAGACTCGGTTGTCCGGAGATTG	XbaI	
pSR6-ORF3F	CAGGTCTCGAGCAAACGGAAAACGCATCAAG	XhoI	876
pSR6-ORF3R	*CAGGTTCTAGACAATTAACCCTCACTAAAGGG	XbaI	

The primer name includes the plasmid and the ORF to be amplified. The amplification products were digested with the indicated enzymes (restriction sites are underlined)
* Indicates that the primer was designed inside the $pSKII^+$ vector, in the case that the ORF was truncated or very close to the polylinker site.

(B) Primers used for subcloning *E. coli* homologs *nth* and *rhlE.*

Primer name	Sequence (5´-3´)	Restriction enzyme	PCR length product (bp)
EndF	CAGGTCTCGAGCAGATGCGCTGTTAGGTAG	XhoI	1712
EndR	CAGGTTCTAGAGCTAAGCAATGGCATCATTG	XbaI	
RhllF	CAGGTCTCGAGGCAGGATTATTCATCGCAC	XhoI	1682
RhlR	CAGGTTCTAGACTGATAAGCGTAGCGCATC	XbaI	

Supplementary Table 3. Primers used for subcloning environmental ORFs in plasmid pdr111 in *B. subtilis.*

Primer name	Sequence (5´-3´)	Restriction enzyme	PCR length product (bp)
pdrSR1-ORF2F	CAGGTAAGCTTCGATCACTACCTGAAGGAC	HindIII	2077
pdrSR1-ORF2R	*CAGGTGCTAGCCAATTAACCCTCACTAAAGGG	NheI	
pdrSR4-ORF1F	*CAGGTAAGCTTCGATAAGCTTGATATCGAATTC	HindIII	1028
pdrSR4-ORF1R	*CAGGTGCTAGCCAATTAACCCTCACTAAAGGG	NheI	
pdrSR6-ORF2F	CAGGTAAGCTTCATTATGGCATTCATGCATTC	HindIII	952
pdrSR6-ORF2R	CAGGTGCTAGCCTCGGTTGTCCGGAGATTG	NheI	
pdrSR7-ORF1F	*CAGGTAAGCTTCGATAAGCTTGATATCGAATTC	HindIII	1239
pdrSR7-ORF1R	*CAGGTGCTAGCCAATTAACCCTCACTAAAGGG	NheI	

Supplementary Table 4. Sequences distribution and comparison of the OPUs diversity (H´), dominance (D) and richness (Chao-1) indexes. (RB, rhizosphere bacterial sample; RA, rhizosphere archaeal sample; BB, brine bacterial sample; BA, brine archaeal sample).

Sample	Seqs	OTUs	OPUs	Index		
				H´	D	Chao-1
**RB**	2495	822	188	4.5	0.02	221.5
**RA**	1140	32	13	2.0	0.2	13
**BB**	1072	60	13	1.8	0.2	12
**BA**	1080	56	13	2.0	0.2	13
**Total**	5780	970	226	-	-	-
**Mean**	1445	242.5	56.5	2.6	0.16	64.9
**Dev**	700.7	386.5	87.7	1.3	0.09	104.4

Supplementary Table 5. Bacteria and Archaea OPUs and their relative abundances for rhizosphere and brine samples from Es Trenc, Mallorca. *Rhiz= rhizosphere. Similarity percentage is indicated in Domain column.

OPU	Bacteria	Rhiz*	Brine
1	*Alphaproteobacteria Xanthobacteraceae Xanthobacteraceae*>94 <98	1.48	0.00
2	*Alphaproteobacteria* Uncultured *Xanthobacteraceae* >94	0.12	0.00
3	*Alphaproteobacteria* Uncultured *Alphaproteobacteria* >81 <99	1.80	0.00
4	*Alphaproteobacteria Hyphomicrobiaceae* *Hyphomicrobium nitrativorans* JX13369 >92 <95	0.20	0.00
5	*Alphaproteobacteria Hyphomicrobiaceae Devosia* spp. >90 <95	0.56	0.00
6	*Alphaproteobacteria Methyloceanibacter caenitepidi* AB794104 >96 <97	0.08	0.00
7	*Alphaproteobacteria Rhizobiales*>90 <95	0.20	0.00
8	*Alphaproteobacteria* Uncultured *Rhizobiales* CU923145 82	0.04	0.00
9	*Alphaproteobacteria Phyllobacteriaceae Aquamicrobium aestuarii* GU199003 >96	0.04	0.00
10	*Alphaproteobacteria Phyllobacteriaceae Hoeflea halophila* GU564401 >99	0.04	0.00
11	*Alphaproteobacteria Aurantimonadaceae Aurantimonadaceae* >97 <99	0.08	0.00
12	*Alphaproteobacteria Aurantimonadaceae Martelella endophytica* HM800924 >93	0.92	0.00
13	*Alphaproteobacteria Alphaproteobacteria* >92 <97	0.20	0.00
14	*Alphaproteobacteria Rhodobacteraceae Labrenzia suaedae* GU322907 >95 <97	0.28	0.00
15	*Alphaproteobacteria Rhodobacteraceae Tropicimonas* sp. >93 <96	0.24	0.00
16	*Alphaproteobacteria Rhodobacteraceae Palleronia marisminoris* AY926462 >96 <97	0.20	0.00
17	*Alphaproteobacteria Rhodobacteraceae Maribius* sp. >95 <98	0.36	0.00
18	*Alphaproteobacteria Rhodobacteraceae Rubellimicrobium* sp. JF139663 >99	0.04	0.00
19	*Alphaproteobacteria Rhodobacteraceae* Uncultured *Rhodobacteraceae* KF500634 >94 <97	0.12	0.00
20	*Alphaproteobacteria Rhodobacteraceae Roseivivax pacificus* KC018453 >94 <97	0.16	0.00
21	*Alphaproteobacteria Rhodobacteraceae Pseudoruegeria haliotis* KC196070 >94	0.20	0.00
22	*Alphaproteobacteria Rhodobacteraceae* Uncultured *Rhodobactearaceae*>86 <96	2.40	0.00
23	*Alphaproteobacteria. Rhodobacteraceae*	0.00	5.60

	Uncultured *Sediminimonas* AF513933 >0.98		
24	*Alphaproteobacteria Caulobacteraceae Caulobacter* spp. >95 <97	0.12	0.00
25	*Alphaproteobacteria* Uncultured *Alphaproteobacteria* GQ262926 >94	0.04	0.00
26	*Alphaproteobacteria Swingsia samuiensis* AB786666 92	0.04	0.00
27	*Alphaproteobacteria Rhodospirillaceae Thalassobaculum* spp. >87 <98	0.72	0.00
28	*Alphaproteobacteria Rhodospirillaceae Tistlia consotensis* CBKU010000188 >87 <97	0.80	0.00
29	*Alphaproteobacteria Rhodospirillaceae* Uncultured *Rhodospirillaceae*>87 <97	0.36	0.00
30	*Alphaproteobacteria Rhodospirillaceae* Uncultured *Rhodospirillaceae*>87 <93	0.32	0.00
31	*Alphaproteobacteria Rhodospirillaceae Defloviicoccus* spp. AJ519652 >93 <96	0.04	0.00
32	*Alphaproteobacteria Rhodospirillaceae* Uncultured *Rhodospirillaceae* >93	0.04	0.00
33	*Alphaproteobacteria Rhodospirillaceae Rhodospirillaceae* AACY023776576>86 <98	0.64	0.00
34	*Alphaproteobacteria. Rhodospirillaceae.* Uncultured *Limimonas* EF105687 >0.98	0.00	12.59
35	*Alphaproteobacteria. Rhodospirillaceae. Limimonas halophila* JN605361 0.95	0.00	1.40
36	*Alphaproteobacteria. Rhodospirillaceae.* Uncultured *Rhodovibrio* EF105852 >0.92	0.00	9.79
37	*Alphaproteobacteria* Uncultured *Alphaproteobacteria* >86 <96	0.36	0.00
38	*Alphaproteobacteria Rickettsiales*>77 <97	0.28	0.00
39	*Alphaproteobacteria Erythrobacteraceae Altererythrobacter* spp. >97 <98	1.16	0.00
40	*Alphaproteobacteria Erythrobacteraceae Erythrobacter odishensis* HE680094 98	0.12	0.00
41	*Alphaproteobacteria Erythrobacteraceae* Uncultured *Altererythrobacter* FJ670864 98	0.08	0.00
42	*Alphaproteobacteria Erythrobacteraceae* Uncultured *Altererythrobacter* FJ562158 >94 <99	0.20	0.00
43	*Alphaproteobacteria Sphingomonadaceae Novosphingobium* spp. >92 <96	0.56	0.00
44	*Alphaproteobacteria* Uncultured *Alphaproteobacteria* >84 <95	0.60	0.00
45	*Alphaproteobacteria* Uncultured *Alphaproteobacteria* >84 <95	0.80	0.00
46	*Alphaproteobacteria Pahyllobacteriaceae* Uncultured *Mesorhizobium* DQ125801 98	0.08	0.00
47	*Alphaproteobacteria*. Uncultured DB1-14 JX882755 0.96	0.00	1.40
48	*Betaproteobacteria Comamonadaceae Ramlibacter* spp. >94 <97	0.24	0.00
49	*Betaproteobacteria Comamonadaceae Hydrogenophaga* spp. AB548035 >98	0.04	0.00

50	Betaproteobacteria Comamonadaceae Uncultured Comamonadaceae >96 <98	0.16	0.00
51	*Betaproteobacteria Rhodocyclaceae Zooglea ramigera* X74913 98	0.08	0.00
52	Betaproteobacteria Nitrosomonadaceae Uncultured Nitrosomonadaceae >90 <98	0.08	0.00
53	*Betaproteobacteria Nitrosomonadaceae Nitrosomonas* sp. Nm143 AY123794 98	0.04	0.00
54	*Betaproteobacteria Nitrosomonadaceae Thiobacter* spp. KF287738 >93 <96	0.20	0.00
55	*Betaproteobacteria Methylophilaceae Methylobacillus flagellatus* KC854922 97	0.08	0.00
56	*Gammaproteobacteria Xanthomonadaceae* *Pseudoxanthomonas dokdonensis* DQ178977 >90	0.04	0.00
57	Gammaproteobacteria Uncultured Xanthomonadales >91 <99	0.68	0.00
58	*Gammaproteobacteria Xanthomonadaceae Rehaibacterium* spp. >91 <100	0.12	0.00
59	*Gammaproteobacteria Ferrimonadaceae Ferrimonas balearica* X93021 >88	0.04	0.00
60	*Gammaproteobacteria Steroidobacter* spp. >90 <98	0.12	0.00
61	*Gammaproteobacteria* RCP1-48 >93 <98	1.60	0.00
62	*Gammaproteobacteria Thiohalomonas* spp. 99	0.04	0.00
63	*Gammaproteobacteria Chromatiaceae Nitrosococcus sp. JN85488 >99*	0.04	0.00
64	*Gammaproteobacteria* SC3-20 FJ793190 93	0.24	0.00
65	*Gammaproteobacteria Plasticicumulans* EU735630 98	0.04	0.00
66	*Gammaproteobacteria Ectothiorhodospiraceae* Uncultured *Ectothiorhodospiraceae* >91 <98	0.52	0.00
67	*Gammaproteobacteria Ectothiorhodospiraceae Ectothiorhodospiraceae*>88 <95	0.08	0.00
68	*Gammaproteobacteria. Ectothiorhodospiraceae*. Uncultured *Aquisalimonas*a FJ152948 0.97	0.00	1.40
69	*Gammaproteobacteria Chromatiales* spp. >93 <98	0.52	0.00
70	*Gammaproteobacteria* H0C36 HQ800864 98	1.28	0.00
71	Gammaproteobacteria Halomonadaceae Halomonas spp. >91 <99	0.32	0.00
72	*Gammaproteobacteria Alteromonadaceae Marinobacter persicus* HQ433441 95	0.04	0.00
73	*Gammaproteobacteria Alteromonadaceae* Uncultured *Alteromonadaceae*>89 <95	0.80	0.00
74	*Gammaproteobacteria Sacharospirillaceae* Uncultured *Sacharospirilla* GU444084 93	0.24	0.00

75	Gammaproteobacteria Uncultured *Gammaproteobacteria*>84 <97	0.60	0.00
76	*Gammaproteobacteria Pseudomaricurvus* sp. >88 <95	1.12	0.00
77	*Gammaproteobacteria* Uncultured *Gammaproteobacteria* 98	0.08	0.00
78	*Gammaproteobacteria Methylohalomonas* sp. HQ397439 96	0.24	0.00
79	*Gammaproteobacteria Methylobacterium* spp. 96	0.04	0.00
80	*Gammaproteobacteria Methylohalomonas* sp. HQ397437 97	0.04	0.00
81	*Gammaproteobacteria* ARKICE-90 JQ425960 >89 <98	0.08	0.00
82	*Gammaproteobacteria Salinisphaera* sp. HQ397443 90	0.08	0.00
83	*Gammaproteobacteria Chromatiaceae* Uncultured *Nitrosococcus* sp. FM553634 93	0.04	0.00
84	*Gammaproteobacteria Pseudomonadaceae Pseudomonas* sp. EU335241 91	0.04	0.00
85	*Gammaproteobacteria* KCM-B-112 HQ397391 >87 <97	0.08	0.00
86	*Deltaproteobacteria* Uncultured *Deltaproteobacteria*>92 <96&	0.24	0.00
87	*Deltaproteobacteria* Uncultured *Myxococcales*>82 <97	0.72	0.00
88	*Deltaproteobacteria* Uncultured *Myxococcales* HQ16681 93	0.08	0.00
89	*Deltaproteobacteria* Uncultured *Sandaracinaceae* >87 <96	0.40	0.00
90	*Deltaproteobacteria Sorangiineae* Uncultured *Sorangiineae*>93 <99	2.93	0.00
91	*Deltaproteobacteria Nannocystaceae Nannocystis* spp. >88 <97	0.88	0.00
92	*Deltaproteobacteria Haliangianceae Haliangium* sp. >88 <95	0.72	0.00
93	*Deltaproteobacteria Anaeromyxobacter* spp. 98	0.12	0.00
94	*Deltaproteobacteria Cystobacteraceae Cystobacter* spp. >94 <98	0.44	0.00
95	*Deltaproteobacteria Cystobacteraceae* Uncultured *Cystobacteraceae* 94	1.84	0.00
96	*Deltaproteobacteria Cystobacteraceae Archangium* sp. EF019058 92	0.04	0.00
97	*Deltaproteobacteria Cystobacteraceae* Uncultured *Cystobacteraceae* >94 <98	0.12	0.00
98	*Deltaproteobacteria* GR-WPP3-58 >80 <96	0.36	0.00
99	*Deltaproteobacteria Bdelovibrionaceae Bdelovibrionaceae* 94	0.12	0.00
100	*Deltaproteobacteria Desulfovibrionales*>84 <86	0.16	0.00
101	*Deltaproteobacteria. Desulfohalobiaceae. Desulfovermiculus* HQ425217 >0.93<0.97	0.00	4.20
102	*Deltaproteobacteria.* Uncultured GR-WP33-58 JX881535 >0.90<0.99	0.00	43.38

103	*Lentisphaerae*. Uncultured *Oligosphaerales* JX885062 >0.98	0.00	1.40
104	*Gemmatimonadetes* BD2-11 >82 <96	3.73	0.00
105	*Gemmatimonadetes Gemmatimonadetes* >84 <96	1.12	0.00
106	*Nitrospirae Nitrospiraceae Leptospirillum* sp. HQ672875 85	0.16	0.00
107	*Deinococcus-Thermus Deinococcaceae Deinococcus* spp. >83 <93	0.60	0.00
108	*Planctomycetes Planctomycetaceae* Uncultured *Planctomyces* >80 <96	3.69	0.00
109	*Planctomycetes Planctomycetaceae* Uncultured *Rhodopirellula*>89 <95	0.28	0.65
110	*Planctomycetes Planctomycetaceae* Uncultured *Pirellula* JN494200 >94 <95	0.12	0.00
111	*Planctomycetes Planctomycetaceae Blastopirellula cremea* JF78733 >88 <90	0.20	0.00
112	*Planctomycetes Planctomycetaceae* Uncultured *Planctomyces* JF319269 >88 <100	0.12	0.00
113	*Planctomycetes Planctomycetaceae* Uncultured *Planctomyces*>78 <95	0.36	0.00
114	Planctomycetes Phycisphaeraceae Uncultured Phycisphaera >75 <93	1.12	0.00
115	*Planctomycete*s Uncultured *Planctomycetes*>79 <92	0.60	0.00
116	Candidate division BRC1 92	0.04	0.00
117	*Verrucomicrobia Opitutaceae Opitutus* spp. EF516121 >81 <96	1.84	0.00
118	Candidate division TM7 >78 <88	0.60	0.00
119	Candidate division 0D1 AY532577 >78 <87	0.36	0.00
120	*Chloroflexi Ardenticatena maritima* AB576167 >74 <83	5.01	0.00
121	*Chloroflexi* Uncultured *Chloroflexi*>79 <97	0.96	0.00
122	*Spirochaetes Spirochaetaceae Sphirochaeta* sp. JN523325 >91	0.44	0.00
123	*Acidobacteria Acidobacteriaceae* Uncultured *Acidobacteriaceae* >79 <96	2.08	0.00
124	*Firmicutes Bacillaceae Bacillus thermotolerans* JX261934 >86 <94	0.56	0.00
125	*Firmicutes Bacillaceae Bacillus halosaccharovorans* HQ433447 >99	3.25	0.00
126	*Firmicutes Bacillaceae Paenibacillus* spp. >92 <97	0.36	0.00
127	*Firmicutes Bacillaceae Tumebacillus flagellatus* JQ421297 >88 <96	0.20	0.00
128	*Firmicutes Bacillaceae* Uncultured *Bacillaceae*>87 <96	0.52	0.00
129	*Firmicutes Planococcaceae Bhargavaea ullalensis* JX144975 >99	0.08	0.00
130	*Firmicutes Planococcaceae Sporosarcina saromensis* AB243859 >94 <100	0.40	0.00

131	*Firmicutes Planococcaceae Paenisporosarcina macmudoensis AJ514408 >97*	0.04	0.00
132	*Firmicutes Planococcaceae Planomicrobium* spp. >95 <97	0.16	0.00
133	*Firmicutes Planococcaceae Bhargavaea* spp. >85 <92	0.36	0.00
134	*Firmicutes Sporolactobacillaceae Sinobaca qinghaiensis* DQ168584 >89	0.04	0.00
135	*Firmicutes Erysipelotrichaceae* Clostridium XVIII >96	0.08	0.00
136	*Firmicutes* Uncultured *Firmicutes* >88 <96	0.20	0.00
137	*Cyanobacteria Nostocaceae Anabaena cylindrica* AF091150 >95	0.08	0.00
138	*Cyanobacteria* Family II *Rivularia* sp. HF678513 >95	0.12	0.00
139	*Cyanobacteria* Family I. *Microcoleus* sp. EF654070 >96	0.32	0.00
140	*Cyanobacteria* Uncultured *Cyanobacteria*>81 <98	0.20	0.00
141	*Fibrobacteres Fibrobacterace*e Uncultured *Fibrobacteraceae* >74 <93	0.16	0.00
142	*Bacteroidetes. Cryomorphaceae.* Uncultured *Owenweeksia* HM127168 0.965	0.00	1.40
143	*Bacteroidetes.*Uncultured *Chitinophagaceae* JX882395 >0.92<0.98	0.00	12.59
144	*Bacteroidetes. Rhodohermaceae. Salinibacter* sp. >0.94<0.999	0.20	4.20
145	*Bacteroidetes Rhodothermaceae* Uncultured *Rhodothermaceae* >89 <98	0.68	0.00
146	*Bacteroidetes Sphingobacteriales Fodinibius salinus* HM153810 >86 <96	1.64	0.00
147	*Bacteroidetes Sphingobacteriales Alliifodinibius sediminis* JQ923476 >99	0.04	0.00
148	*Bacteroidetes Sphingobacteriales Fodinibius* spp. >86 <97	0.92	0.00
149	*Bacteroidete*s Uncultured *Bacteroidetes*>87 <92	0.80	0.00
150	*Chlorobi Ignavibacteriaceae Ignavibacterium* spp. >93	0.28	0.00
151	*Bacteroidetes* Marine Bacterium JK1007 JX050172 >97	0.04	0.00
152	*Bacteroidetes Cyclobacteriaceae Mongoliicoccus alkaliphilus* HE996970 95	0.04	0.00
153	*Bacteroidetes Cytophagaceae* Uncultured *Cytophagaceae*>87 <96	3.61	0.00
154	*Bacteroidetes* Uncultured *Cytophagales*>85 <98	0.52	0.00
155	*Bacteroidetes* Uncultured *Cytophagales* >88 <97	0.40	0.00
156	*Bacteroidetes Cytophagales* Uncultured *Cytophagales*>78 <97	1.28	0.00
157	*Bacteroidetes Cytophagaceae Pontibacter odishensis* HE681883 >93	0.04	0.00
158	*Bacteroidetes Flammeovirgaceae Marinoscillum luteum* HM16878 >94 <98	0.20	0.00

159	*Bacteroidetes Flammeovirgaceae Fulvivirga kasyanovii* >88 <98	0.12	0.00
160	*Bacteroidetes Flammeovirgaceae* Candidatus *Amoebophilus asiaticus* 5a2 SP001102 >94	0.08	0.00
161	*Bacteroidetes Flavobacteriaceae Salegentibacter* sp. RV2 GQ365193 >88 <91	0.92	0.00
162	*Bacteroidetes Flavobacteriaceae Gramella flava* JX397931 >98	0.08	0.00
163	*Bacteroidetes Flavobacteriaceae Salinimicrobium* sp. >93	0.04	0.00
164	*Bacteroidetes Flavobacteriaceae Gelidibacter* spp. >91 <97	0.48	0.00
165	*Bacteroidetes Flavobacteriaceae Sinomicrobium oceani* JQ352762 >90	0.24	0.00
166	*Bacteroidetes Flavobacteriaceae Muricauda* spp. >97	0.04	0.00
167	*Bacteroidetes Flavobacteriaceae Flavobacteriaceae*>80 <88	0.92	0.00
168	*Bacteroidetes Cryomorphaceae Owenweeksia* sp. KC331461 >90	0.04	0.00
169	*Bacteroidetes Sapropiraceae Lewinella* spp. >81 <95	0.12	0.00
170	*Actinobacteria Microbacteriaceae Microbacteriaceae* >85 <92	0.72	0.00
171	*Actinobacteria Vibrionaceae Vibrio ponticus* AJ630103 >84 <86	0.16	0.00
172	*Actinobacteria* Uncultured *Actinobacteria*>79 <90	2.97	0.00
173	*Actinobacteria* 480-2 >86 <97	2.20	0.00
174	*Actinobacteria Micromonosporaceae Pilimelia* sp. HM445002 95	0.08	0.00
175	*Actinobacteria Nitriliruptoraceae Nitriliruptor* spp. >88 <98	1.76	0.00
176	*Actinobacteria Euzebyaceae Euzebyaceae* >93 <98	2.04	0.00
177	*Actinobacteria* Uncultured *Actinobacteria*>90 <98	1.36	0.00
178	*Actinobacteria Aquihabitans daechungensis* JN033775 94	0.56	0.00
179	*Actinobacteria* Sva0996 GQ472831 >90 < 96	0.20	0.00
180	*Actinobacteria Acidimicrobiales*>93 < 99	0.96	0.00
181	*Actinobacteria Acidimicrobiaceae Ilumatobacter* spp. >90 <99	1.32	0.00
182	*Actinobacteria Streptomycetaceae Streptomyces* spp. >97 <999	0.28	0.00
183	*Actinobacteria Nocardiaceae Nocardioides* spp. >98 <99	0.24	0.00
184	*Marmoricola* sp. KC820854 98	0.04	0.00
185	*Actinobacteria Nocardiaceae Kribbella albertanoniae* 99	0.04	0.00

186	*Actinobacteria Nocardiaceae Nocardia* spp. 93	0.04	0.00
187	*Actinobacteria Nocardiopsaceae Haloactinospora alba* DQ923130 99	0.32	0.00
188	*Actinobacteria Glycomycetaceae* Uncultured *Glycomycetaceae* GQ263395 >81 <91	0.52	0.00
189	*Actinobacteria Pseudonocardiaceae Amycolatopsis salitolerans* FJ606836 99	0.16	0.00
190	*Actinobacteria Pseudonocardiaceae Amycolaptosis* spp 99	0.04	0.00
191	*Actinobacteria* Uncultured *Actinobacteria* 98	0.40	0.00
192	*Actinobacteria Actinopolysporaceae Actinopolyspora* spp. >98 < 999	0.08	0.00
193	*Actinobacteria Geodermatophilaceae Blastococcus* spp. >98 < 999	0.56	0.00
194	*Actinobacteria* Uncultured *Actinobacteria*>98 < 999	0.72	0.00
195	*Actinobacteria Micrococcaceae Kocuria* spp. 99	0.04	0.00
196	*Actinobacteria Micrococcaceae Arthrobacter* spp. 97	0.04	0.00
197	*Actinobacteria Micrococcineae Luteimicrobium* spp. 99	0.44	0.00
198	*Actinobacteria Microbacteriaceae Glaciihabitans tibetensis* KC256953 99	0.68	0.00
199	*Actinobacteria Intransporanginaceae Aquipuribacter* sp. EU930868 99	0.12	0.00
200	*Actinobacteria Corynebacteriaceae Corynebacterium matruchotii* X82065 93	0.04	0.00
	**Total Bacteria**	100.0	100.0
	**Archaea**		
201	*Euryarchaeota. Halobacteriaceae. Halococcus dombrowskii* AJ420376 >0.98	1.32	0.00
202	*Euryarchaeota. Halobacteriaceae. Halococcus hamelinensis* DQ017835 >0.95<0.97	6.58	0.00
203	*Euryarchaeota. Halobacteriaceae. Halococcus* spp. >0.93 <0.95	1.32	0.00
204	Euryarchaeota. Halobacteriaceae. Haladaptatus paucihalophilus DQ344973 >0.98	27.63	0.00
205	*Euryarchaeota. Halobacteriaceae.* Uncultured *Haladaptatus* HQ400419 >0.94<0.96	25.00	0.00
206	*Euryarchaeota. Halobacteriaceae. Halobonum* JN714431 >0.99	14.47	0.00
207	*Euryarchaeota. Halobacteriaceae. Halobonum* HQ400558 >0.97	1.32	0.00
208	*Euryarchaeota. Halobacteriaceae. Halomarina oriensis* AB519798 0.923	0.00	3.70
209	Euryarchaeota. Halobacteriacea. *Halorubrum aquaticum* AM268115 0.94	0.00	14.81
210	*Euryarchaeota. Halobacteriacea. Halorubrum orientale* AM235789 >0.99	0.00	37.04
211	*Euryarchaeota. Halobacteriacea*. Uncultured *Halorubrum* HQ157591 0.975	0.00	1.85

212	*Euryarchaeota. Halobacteriacea. Halorubrum xinjiongense* AY510707 >0.98	0.00	5.56
213	*Euryarchaeota. Halobacteriacea. Halorubrum* spp >0.99	0.00	1.85
214	*Euryarchaeota. Halobacteriacea. Haloferax mediterranei* D11107 >0.98	1.32	0.00
215	*Euryarchaeota. Halobacteriacea. Halopelagius fulvigenes* JQ996497 0.94	3.95	0.00
216	*Euryarchaeota. Halobacteriacea. Halopelagius inordinatus* EU887284 0.92	6.58	0.00
217	*Euryarchaeota. Halobacteriacea. Halobellus salinus* HQ451075 >0.94<0.99	0.00	5.56
218	*Euryarchaeota. Halobacteriacea. Halobellus clavatus* GQ282620 >0.98	0.00	1.85
219	Euryarchaeota.*Halobacteriacea*. Uncultured *Halobellus* FN391236 >0.98	1.32	0.00
220	*Euryarchaeota. Halobacteriacea.* Uncultured *Haloquadratum* CU467219 >0.99	0.00	16.67
221	*Euryarchaeota. Halobacteriaceae.* Uncultured *Haloarcula* HE604439 >0.98	0.00	1.85
222	*Euryarchaeota. Halobacteriaceae*. Uncultured *Haloarcula* HQ400420 0.978	0.00	1.85
223	*Euryarchaeota. Halobacteriaceae. Halonotiu*s AM947464 >0.99	0.00	3.70
224	*Euryarchaeota. Halobacteriaceae* CU467243 >0.98< 0.94	0.00	3.70
225	*Euryarchaeota. Methanosarcinaceae.* Uncultured *Methanosarcina* EU420698 >0.98	3.95	0.00
226	*Euryarchaeota. Methanoregulaceae. Methanolinea mesophila* AB447467 >0.98	5.26	0.00
	**Total Archaea**	100.0	100.0

Supplementary Table 6. Salt resistance screening of the libraries constructed with DNA from brine (LSR-b) and rhizosphere (LSR-r) samples.

Library	No. of clones	Average insert size (Kb) [a]	Library size (Mb)	No. of NaCl resistant clones[b]
Brine	236,250	2.9	685	3
Rhizosphere	192,000	3	576	5
Total	428,250	2.95	1,261	8

[a] Estimated from restriction digest analysis of 16 random clones using EcoRI and XbaI enzymes.
[b] Number of NaCl resistant clones recovered after retransformation and fragment length polymorphism analysis.

Supplementary Table 7. Putative functions of the translated ORFs based on protein family domains (Pfam).

Plasmid	ORF	Pfam family (E value)
pSR1	ORF1	Peptidase_S9 (1.9e-16)
	ORF2	UvrD-helicase (2.8e-25)
pSR2	ORF1	No matches
	ORF2	No matches
pSR3	ORF1	Cell adhesion related domain found in bacteria CARDB (1.1e-10)
	ORF2	No matches
pSR4	ORF1	HhH-GPD superfamily base excision DNA repair protein (1.3e-20)
pSR5	ORF1	VWA (von Willebrand factor type A domain) (1e-10)
	ORF2	No matches
pSR6	ORF1	OmpA (4.4e-18)
	ORF2	MIP (Major intrinsic protein) (3.8e-14)
	ORF3	Sulfatase (1.4e-25)
pSR7	ORF1	DEAD (DEAD/DEAH box helicase) (2.4e-11)
pSR8	ORF1	H_PPase (Inorganic H+ pyrophosphatase) (1.1e-56)

1.2. Supplementary Figures

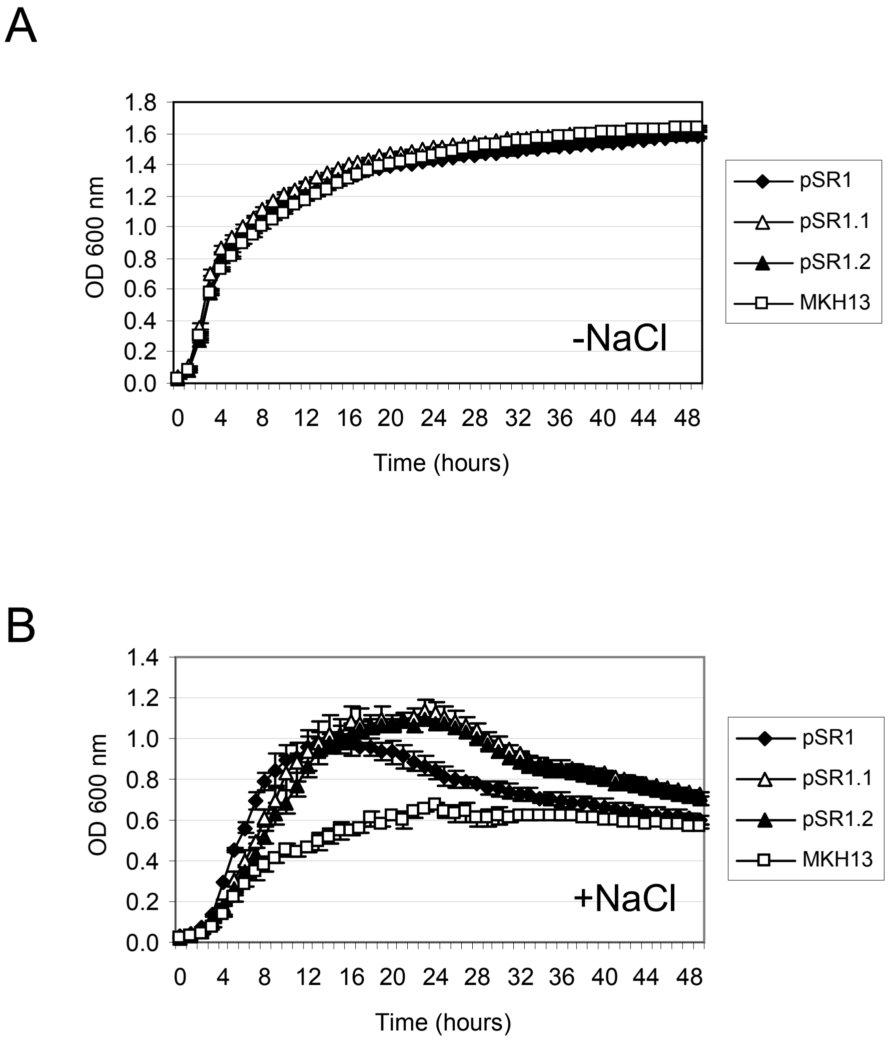

Supplementary Figure 1. Growth curve of *E. coli* MKH13 cells carrying pSR1, pSR1-*orf1*, pSR1-*orf2* and MKH13-pSKII^{+} in LB broth (A) and LB broth supplemented with 3% NaCl (B).

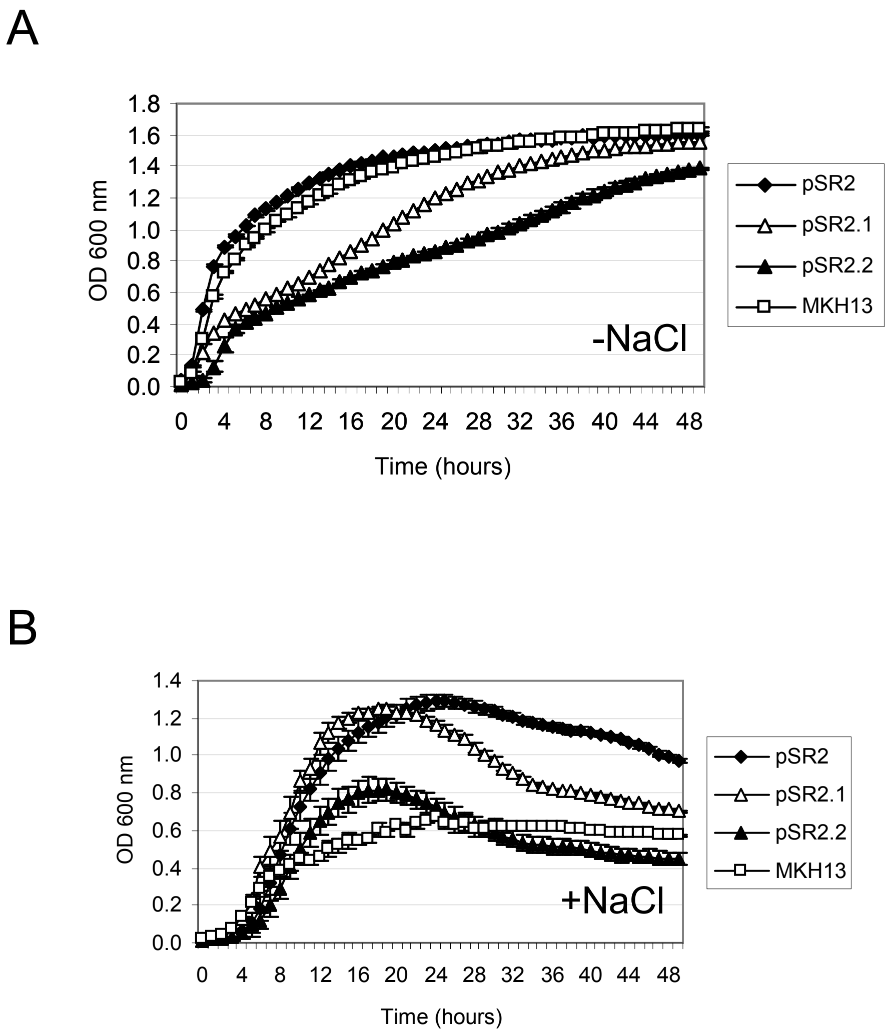

Supplementary Figure 2. Growth curve of *E. coli* MKH13 cells carrying pSR2, pSR2-*orf1*, pSR2-*orf2* and MKH13-pSKII^{+} in LB broth (A) and LB broth supplemented with 3% NaCl (B).

A

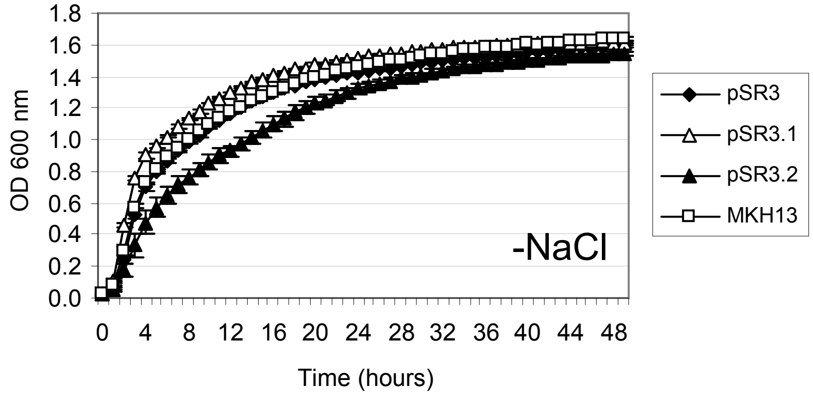

B

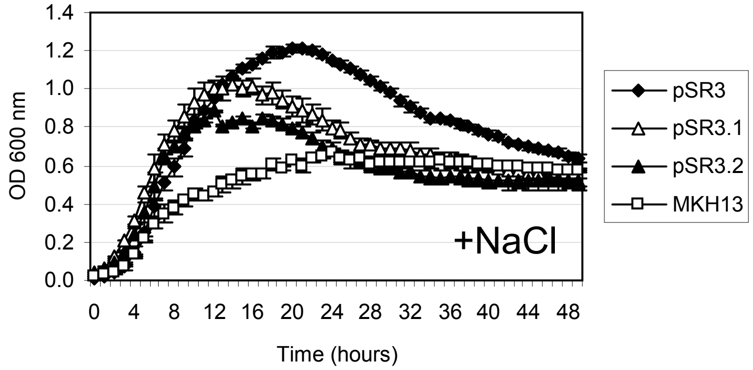

Supplementary Figure 3. Growth curve of *E. coli* MKH13 cells carrying pSR3, pSR3-*orf1*, pSR3-*orf2* and MKH13-pSKII^{+} in LB broth (A) and LB broth supplemented with 3% NaCl (B).

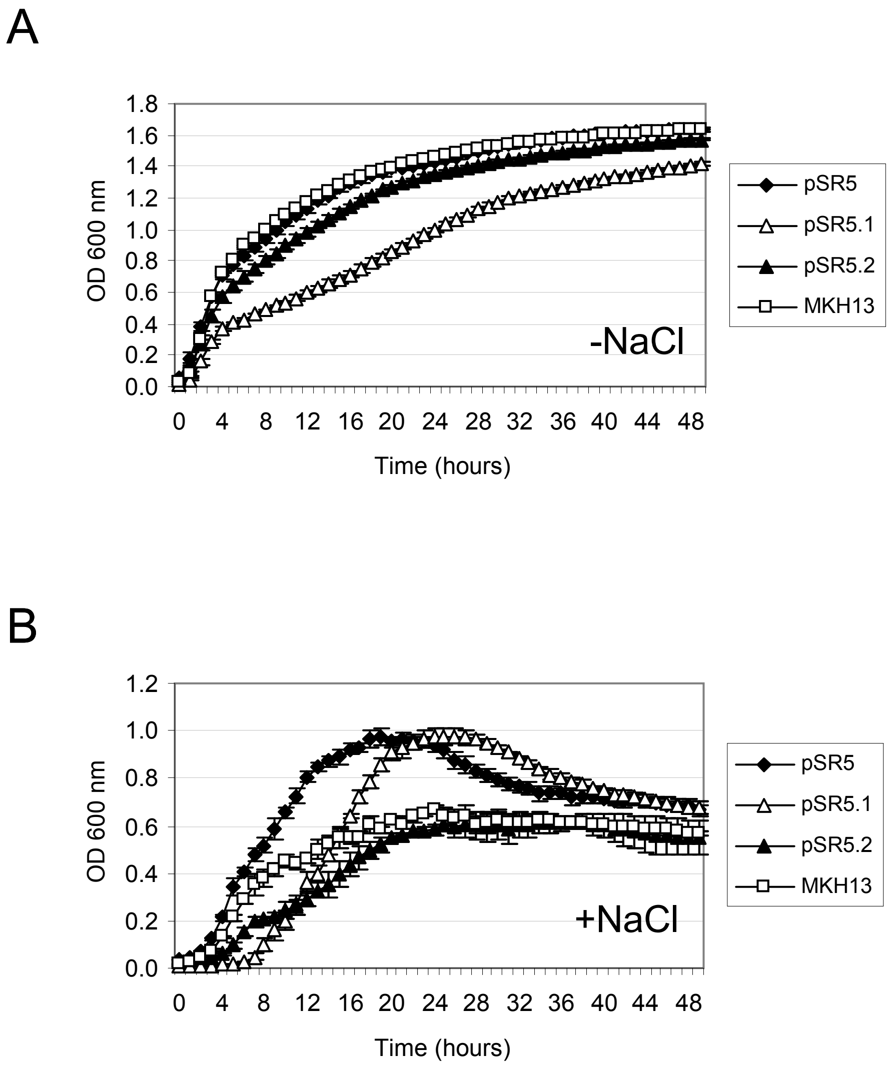

Supplementary Figure 4. Growth curve of *E. coli* MKH13 cells carrying pSR5, pSR5-*orf1*, pSR5-*orf2* and MKH13-pSKII$^+$ in LB broth (A) and LB broth supplemented with 3% NaCl (B).

A

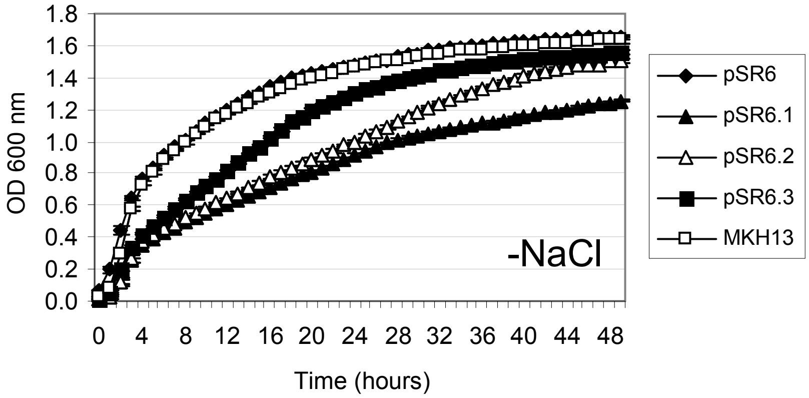

B

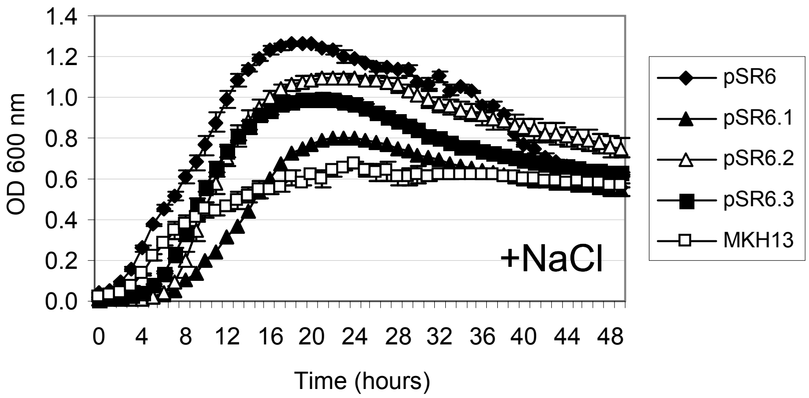

Supplementary Figure 5. Growth curve of *E. coli* MKH13 cells carrying pSR6, pSR6-*orf1*, pSR6-*orf2*, pSR6-*orf3* and MKH13-pSKII$^+$ in LB broth (A) and LB broth supplemented with 3% NaCl (B).
